# Periaqueductal grey and spinal cord pathology contribute to pain in Parkinson’s disease

**DOI:** 10.1038/s41531-023-00510-3

**Published:** 2023-04-26

**Authors:** Yazead Buhidma, Carl Hobbs, Marzia Malcangio, Susan Duty

**Affiliations:** King’s College London, Institute of Psychiatry, Psychology & Neuroscience, Wolfson Centre for Age-Related Diseases, Guy’s Campus, London, SE1 1UL UK

**Keywords:** Neurological manifestations, Parkinson's disease

## Abstract

Pain is a key non-motor feature of Parkinson’s disease (PD) that significantly impacts on life quality. The mechanisms underlying chronic pain in PD are poorly understood, hence the lack of effective treatments. Using the 6-hydroxydopamine (6-OHDA) lesioned rat model of PD, we identified reductions in dopaminergic neurons in the periaqueductal grey (PAG) and Met-enkephalin in the dorsal horn of the spinal cord that were validated in human PD tissue samples. Pharmacological activation of D_1_-like receptors in the PAG, identified as the DRD5^+^ phenotype located on glutamatergic neurons, alleviated the mechanical hypersensitivity seen in the Parkinsonian model. Downstream activity in serotonergic neurons in the Raphé magnus (RMg) was also reduced in 6-OHDA lesioned rats, as detected by diminished c-FOS positivity. Furthermore, we identified increased pre-aggregate α-synuclein, coupled with elevated activated microglia in the dorsal horn of the spinal cord in those people that experienced PD-related pain in life. Our findings have outlined pathological pathways involved in the manifestation of pain in PD that may present targets for improved analgesia in people with PD.

## Introduction

Pain in Parkinson’s disease is one of the most debilitating non-motor symptoms^1^. The pain can be subcategorised as either spontaneous pain associated with Parkinson’s disease (SPPD) or hypersensitivity, manifest as reduced pain thresholds in response to evoked noxious stimulation^2^. Conventional wisdom was that SPPD was driven by motor deficits^3^ but mounting evidence suggests that neither pain occurrence, severity, nor duration are correlated with motor scores^4,5^. Rather, these pain features are significantly correlated with affective and autonomic symptoms that typically occur in the premotor stages of Parkinson’s disease^5^.

Numerous studies have reported a general reduction in electrical, thermal, cold, and mechanical thresholds of Parkinson’s disease patients compared to healthy controls, using quantitative sensory testing (QST)^6–10^. However, this hypersensitivity manifests regardless of whether patients exhibit SPPD or not, as defined by self-reported Visual Analogue Scale, clinical questionnaires, and the McGill Pain Questionnaire^7,9,11–19^. Furthermore, people with Parkinson’s disease that present with SPPD have similar pain thresholds to those patients without SPPD^7–9,20^. This suggests two separate, as yet unknown, mechanisms underlie the hypersensitivity and SPPD symptoms.

Despite the prevalence and impact of pain in Parkinson’s disease, few effective treatments exist. SPPD is often managed through maintaining stable dopaminergic transmission in the brain. Indeed levodopa (L-DOPA) has shown promise against both SPPD and hypersensitivity in Parkinson’s disease patients^9,10,12,15,21–23^. Other efficacious dopaminergic treatments include rotigotine, a DA agonist transdermal patch; safinamide, a monoamine oxidase B inhibitor given in tandem with L-DOPA; and intrajejunal L-DOPA infusion therapy^24^.

SPPD is largely considered a prodromal symptom. During these premotor stages of Parkinson’s disease, Lewy pathology, the characteristic hallmark of Parkinson’s disease comprised of aggregated α-synuclein, is confined to the midbrain and hindbrain regions. The periaqueductal grey (PAG), a key pain processing region in the midbrain, exhibits significant Lewy body and Lewy neurite deposition in early Parkinson’s disease^25,26^ predisposing it to functional deficits. Indeed, functional magnetic resonance imaging data supports the PAG having reduced activity in Parkinson’s disease, which is predicted to reduce pain thresholds^27^. Furthermore, the PAG, particularly the ventrolateral tier (VL-PAG), is the only reported nucleus affected in early Parkinson’s disease that contains dopaminergic antinociceptive circuitry. Most notably, Flores et al.^28,29^ showed that PAG-specific D_1_-signalling was critical for the analgesic effects of opioids, ultimately revealing that D_1_-like receptor stimulation in the VL-PAG brings about antinociception^30,31^. The identity of the downstream neural cell and specific D_1_-like receptor involved, is unknown.

The descending projections from the VL-PAG indirectly modulate ascending nociceptive signals within the dorsal horn of the spinal cord (SC). As in other chronic pain conditions, disruption of these dorsal horn processes may contribute to increased sensitisation to painful stimuli in Parkinson’s disease. Although the SC is known to exhibit α-synuclein pathology at the later stages of Parkinson’s disease^32^, it is not known whether pain-related signalling in the SC is affected in people with Parkinson’s disease.

Unravelling the contribution of VL-PAG and SC signalling to pain in Parkinson’s disease may help find better analgesic targets. Animal models enable investigations of nociceptive behaviour alongside detailed assessment of the contributory neural mechanisms. The most used preclinical model for studying pain in Parkinson’s disease is the unilateral 6-hydroxydopamine (6-OHDA) lesioned rodent. In rats or mice, lesioning the nigrostriatal tract leads to reduced nociceptive thresholds to heat, mechanical, chemical, or cold stimuli^2^. These threshold changes are seen bilaterally, despite the lesion, and motor impairment being unilateral^2^. This reveals that, consistent with the clinical picture, the hypersensitivity is independent of the motor impairment^5^. Moreover, considering hindbrain pain circuitry is known to project to both hemispheres, this bilateral hypersensitivity supports the involvement of dysregulated bilateral supraspinal pain nuclei in the brainstem. Similar to Parkinson’s disease patients^10,15,33,34^, administration of L-DOPA to 6-OHDA lesioned rodents raises mechanical thresholds back to normal^35^. We propose this effect of L-DOPA is achieved through restoration of dopaminergic transmission in extra-nigral, pain-related dopaminergic neurocircuitry at the supraspinal level.

The 6-OHDA rodent model of Parkinson’s disease also exhibits dysregulation in non-dopaminergic pathways involved in pain, notably the opioidergic system. Such findings include bilateral reductions in endogenous opioid peptide, Met-enkephalin (Met-ENK), lower levels of μ-opioid receptors in the SC, and increased windup of wide dynamic range neurons thought to be controlled by endogenous opioid mechanisms^35–37^. Reductions in Met-ENK have also been reported in the CSF of people with Parkinson’s disease^38^ but this has not yet been linked to pain manifestation. The endogenous opioidergic system is regulated by noradrenergic neurons of the locus coeruleus and serotonergic neurons of the rostral ventral medulla (RVM). Of particular interest are the serotonergic neurons of the Raphé Magnus (RMg), which lie within the RVM. Dysfunction of these serotonergic RMg neurons has been noted to drive mechanical and thermal hypersensitivity in Parkinsonian rats^39,40^ but it remains to be seen whether this is mediated via a reduction Met-ENK levels within the SC.

To advance our understanding of the pathophysiology of pain in Parkinson’s disease, and identify potential analgesic targets, we undertook a more systematic and detailed exploration of the 6-OHDA model. We hypothesised that: (1) the hypersensitivity seen in the 6-OHDA model of Parkinson’s disease is caused by dysregulation of descending dopaminergic circuitry within the PAG; (2) pharmacological reinstatement of dopaminergic transmission in the PAG would be antinociceptive; (3) the reduced dopaminergic transmission in the PAG drives a pronociceptive environment within the dorsal horn of the SC through reduced Met-ENK levels, resulting from attenuated serotonergic activity in the RMg; (4) Parkinson’s disease patients exhibit the same pathology in the PAG and SC as seen in the 6-OHDA lesioned rat; and (5) patients with SPPD exhibit an additional pathological feature that may lead to their manifestation of spontaneous pain. We tested these hypotheses by characterising the hypersensitivity in the 6-OHDA rat model of Parkinson’s disease and thus identified key pathological changes in pain-related regions that are affected in early Parkinson’s disease. In addition, we identified the impact of the pathology on pain processing and delineated the pathway involved. We also confirmed the same pathology in those affected by Parkinson’s disease, adding translational significance to our findings.

## Results

### Hemiparkinsonian rats show nociceptive hypersensitivity and dopaminergic cell loss in the PAG

**Fig. 1:**
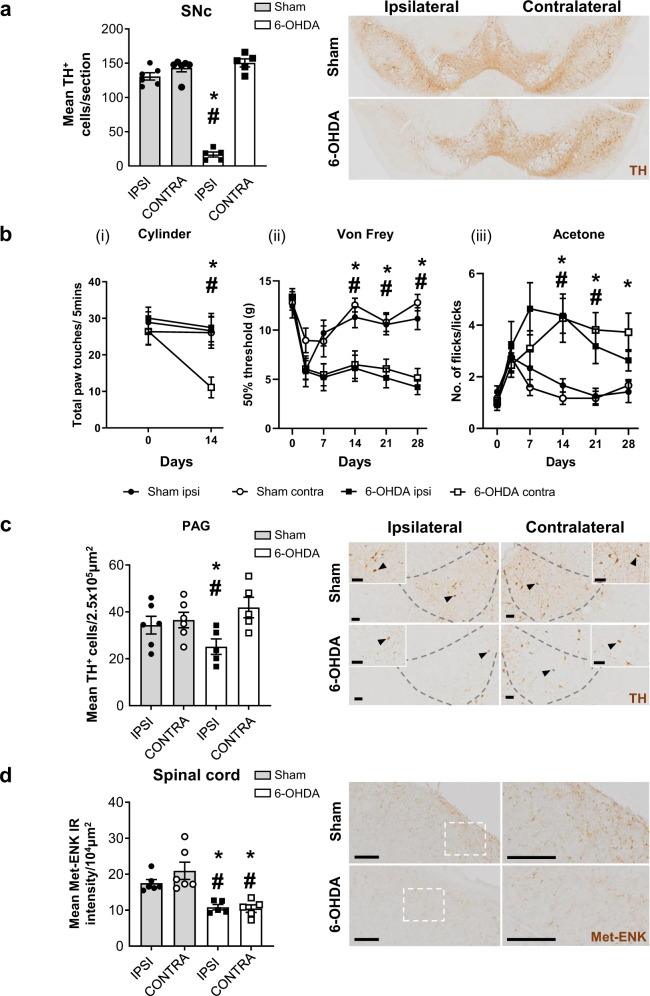
Hemiparkinsonian rats exhibit nociceptive hypersensitivity and dopaminergic cell loss in the VL-PAG. **a** Total mean number of TH^+^ cells in the ipsi- and contra- lateral sides of the substantia nigra pars compacta (SNc; across rostral, medial, and caudal tiers) in sham- (*n* = 6) and 6-OHDA-lesioned (*n* = 5) rats. Representative coronal sections of the caudal substantia nigra of sham and 6-OHDA lesioned rats stained for tyrosine hydroxylase via immunohistochemistry. **b** (i) Total number of paw touches of sham (*n* = 13) and 6-OHDA (*n* = 12) lesioned rats at baseline and 14 days post surgery using the cylinder test (*F* = 44.05, *P* < 0.0001). (ii) Hind paw mechanical thresholds of sham and 6-OHDA lesioned rats from baseline up to 28-days post surgery using the von Frey test (ipsi; *F* = 16.04, *P* = 0.0006; contra; *F* = 22.43, *P* = 0.0001). (iii) Hind paw thresholds to cold stimulation of sham and 6-OHDA lesioned rats from baseline up to 28-days post surgery using the acetone test (ipsi; *F* = 10.28, *P* = 0.0042; contra; *F* = 13.73, *P* = 0.0013). **c** The total mean number of TH^+^ cells in the ipsi- and contralateral ventrolateral tier of the periaqueductal grey (PAG) across rostral, medial, and caudal areas in sham (*n* = 6) and 6-OHDA (*n* = 5) lesioned rats. Representative images of TH stain in the medial PAG of sham and 6-OHDA lesioned rats with higher magnification inserts and arrows indicating representative TH^+^ cells. **d** Immunoreactivity intensity of Met-enkephalin staining in the ispi- and contralateral dorsal horn of the spinal cord in sham and 6-OHDA lesioned rats. Representative images of met-enkephalin IHC stain in the dorsal horn of the spinal cord in sham and 6-OHDA lesioned rats. All data represented as mean ± S.E.M. Behavioural analyses were performed using a two-way ANOVA with a Tukey’s multiple comparisons test. Post-mortem histological analyses were performed using Kruskal–Wallis with Dunn’s multiple comparisons tests, * indicates that *P* < 0.05 comparing sham with 6-OHDA and # indicates that *P* < 0.05 for 6-OHDA ipsi- vs. contralateral. All scalebars = 50 µm.

Post-mortem validation of the Parkinson’s disease model confirmed that intra-MFB 6-OHDA caused significant ablation of TH^+^ cells in the SNc ipsilateral to the injection with no cell loss in sham rats (Fig. 1a). Using the cylinder test, sham rats exhibited no changes in paw touches. 6-OHDA lesioned rats exhibited >50% reduction in contralateral paw touches coupled with no changes in ipsilateral paw touches on day 14 compared to baseline (Fig. 1bi).

Using manual von Frey testing of 50% withdrawal thresholds, sham rats exhibited a transient, bilateral reduction in mechanical thresholds at day 3 post surgery, which returned towards pre-treatment baseline of ~15 g, at day 7 (Fig. 1bii). 6-OHDA-lesioned rats presented bilateral reduced thresholds at day 3 that persisted, reaching significance from day 14 onwards (Fig. 1bii). Similarly, both 6-OHDA and sham rats exhibited a bilateral increase in licking and flicking responses upon application of acetone to the hind paw at 3 days post surgery, with this persisting only in the 6-OHDA rats, reaching significance from day 14 onwards (Fig. 1biii).

Post-mortem analysis revealed a significant reduction in TH^+^ cells in the ipsilateral VL-PAG of the intra-MFB 6-OHDA lesioned rats (Fig. 1c). There was no evidence of noradrenergic dopamine beta-hydroxylase (DβH^+^) cells present in the PAG (Supplementary Fig. 1) confirming the TH^+^ cell loss as dopaminergic. Moreover, the cell loss was primarily restricted to the medial part of the VL-PAG (Supplementary Fig. 1). In the SC, bilateral reductions in intensity of Met-ENK^+^ immunoreactivity were recorded in the superficial laminae of dorsal horn in the L3-L5 lumbar regions (Fig. 1d). In contrast, no significant changes were observed across a wide range of other markers in the SC that could potentially have contributed to the nociceptive changes measured, including NeuN^+^ or Iba1^+^ cell counts, or intensity of GFAP, TPH, CGRP, DYN, GAD65/67 or DβH immunoreactivity (Supplementary Fig. 2). Similarly, no changes were seen in the L3-L5 DRGs using NF200 and CGRP, thus ruling out any potential peripheral neuropathic contribution to the hypersensitivity (Supplementary Fig. 2). Within the other pain-related nuclei, no changes were noted in the hindbrain for DβH + cells in the LC or TPH + cells in the RMg (Supplementary Fig. 2). Within the midbrain, ipsilateral reductions in TH^+^ cells in the rostral ventral tegmental area (VTA) were noted, but with no reductions in TPH^+^ and/or TH^+^ cells in either the dorsal Raphé nuclei (DRN) or A11 region of the hypothalamus, respectively (Supplementary Fig. 2). In summary, the 6-OHDA lesioned rats have a selective ipsilateral dopaminergic cell loss in the PAG along with selective bilateral reductions in Met-ENK levels in the SC.

### Loss of dopaminergic neurons in PAG causes hypersensitivity and reduces spinal cord Met-enkephalin

**Fig. 2:**
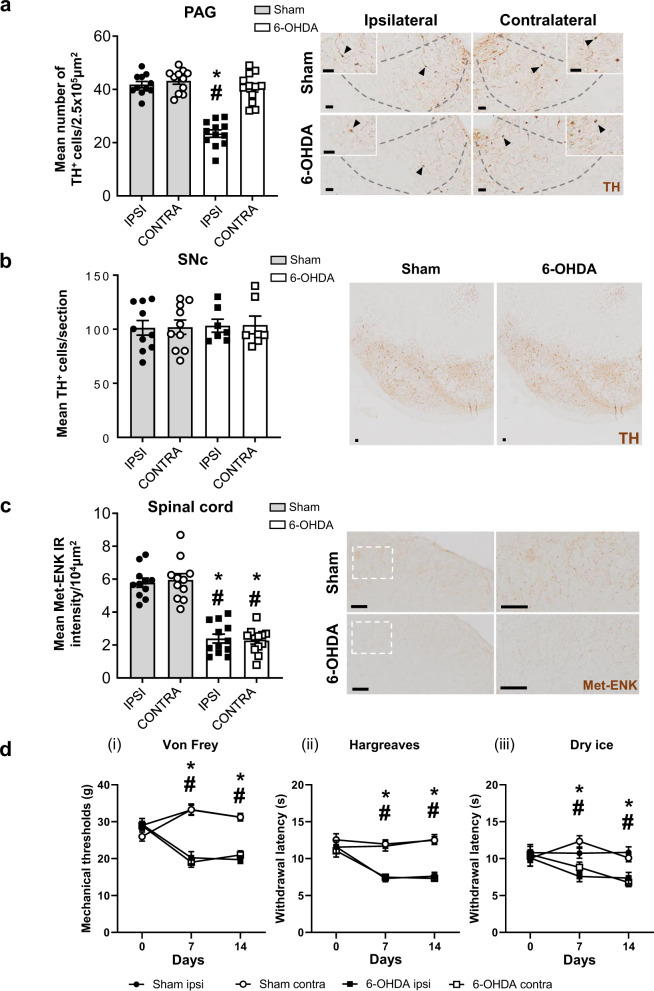
Ablation of dopaminergic neurons in the VL-PAG causes hypersensitivity with accompanied reductions of Met-ENK in the spinal cord. **a** Total number of TH + cells in the ipsi- and contralateral sides of the periaqueductal grey (PAG) in sham- (*n* = 12) and 6-OHDA- (*n* = 12) PAG-lesioned rats. Representative coronal sections of the medial VL-PAG of sham and 6-OHDA lesioned rats stained for tyrosine hydroxylase with higher magnification inserts and arrows indicating representative TH + cells. **b** Total mean number of TH^+^ cells in the ipsi- and contralateral sides of the substantia nigra pars compacta (SNc). Representative coronal TH-stained sections of the caudal SNc from sham abd 6-OHDA lesioned rats. **c** Mean immunoreactivity intensity of Met-ENK in the dorsal horn of the spinal cord in sham and 6-OHDA-PAG-lesioned rats: each image has higher magnification images from within the dotted box. **d** Mechanical and thermal thresholds of sham and 6-OHDA PAG-lesioned rats on baseline, day 7, and day 14 post lesion. (i) Mechanical thresholds (ipsi; *F* = 35.88, *P* < 0.0001; contra; *F* = 58.62, *P* < 0.0001), (ii) heat thresholds (ipsi; *F* = 28.53, *P* < 0.0001; contra; *F* = 46.86, *P* < 0.0001), and (iii) cold thresholds (ipsi; *F* = 5.739, *P* < 0.0265; contra; *F* = 5.867, *P* < 0.025) were measured using the von Frey, Hargreaves, and dry-ice tests, respectively. All data are represented as mean ± SEM. Behavioural analyses were performed using a two-way ANOVA with a Tukey’s multiple comparisons test. Post-mortem histological analyses were performed using Kruskal–Wallis with Dunn’s multiple comparisons tests, * indicates that *P* < 0.05 comparing sham with 6-OHDA and ^#^ indicates that *P* < 0.05 for 6-OHDA ipsi- vs. contralateral. All scalebars = 50 µm.

To investigate the functional impact of chronic reductions in dopaminergic tone in the PAG, we injected 6-OHDA or vehicle into the left VL-PAG of Wistar rats. Post-mortem analysis confirmed that 6-OHDA caused a significant reduction (~50%) in ipsilateral dopaminergic cells within the VL-PAG (Fig. 2a). The loss was most evident in medial and rostral portions of the VL-PAG, where dopaminergic cells were denser (Supplementary Fig. 3). The 6-OHDA PAG-lesion did not induce TH^+^ cell loss in the VTA or DRN (Supplementary Fig. 3), or in the SNc (Fig. 2b), confirming a selective ablation of cells in the VL-PAG, without collateral damage. Moreover, there was no impact on TPH^+^ numbers in the ventral-PAG or DRN (Supplementary Fig. 3), confirming that the lesion exclusively impacted dopaminergic cells of the PAG. Investigation of the spinal cord identified significant bilateral reductions in Met-ENK immunoreactivity of 6-OHDA PAG-lesioned rats (Fig. 2c).

Regarding the nociceptive changes, as expected, sham PAG-lesioned rats exhibited no changes in mechanical thresholds, measured via automated von Frey (100% thresholds remained ~30 g). Similarly, no changes were seen in thermal thresholds, measured by Hargreaves and Dry-ice testing when compared to baseline (Fig. 2d). The 6-OHDA PAG-lesioned rats, however, displayed significant reductions in mechanical thresholds on day 7 and 14 in comparison to baseline and sham animals (Fig. 2di) and had increased response time of paw withdrawals when exposed to the heat and cold (Fig. 2dii, iii). All changes in threshold were evident bilaterally.

Altogether, these findings show that selective loss of dopaminergic cells of the PAG is sufficient to reproduce the sustained bilateral hypersensitivity and bilateral reductions in SC Met-ENK previously seen in the hemiparkinsonian rats.

### Stimulation of D_1_-like receptors in the PAG produces analgesia in Parkinsonian rats

**Fig. 3:**
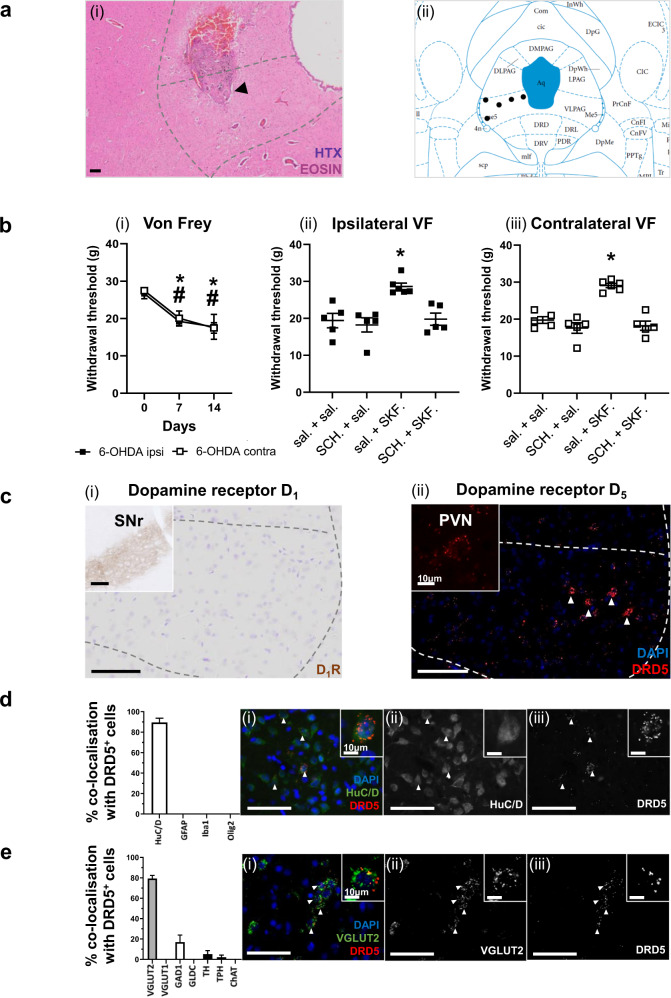
D_1_-like receptor stimulation in the VL-PAG mediates an analgesic effect in Parkinsonian rats through the likely activation of D5 receptors on glutamatergic neurons. **a** Representative image showing (i) haematoxylin and eosin-stained periaqueductal grey (PAG) cannula track mark and arrow indicating correct cannula placement into the VL-PAG, with (ii) the location of the cannulae for each animal superimposed on an image from the rat brain atlas by Paxinos & Watson (2005). **b** Development of mechanical hypersensitivity in 6-OHDA-lesioned hemiparkinsonian rats in both ipsi- and contralateral hind paws using the von Frey at baseline, days 7 and 14 post lesion (*n* = 5; *F* = 24.83, *P* < 0.0001). Mechanical thresholds for the 6-OHDA lesioned rats at 2-weeks post lesion, when given saline + saline, saline + SKF38393, SCH23390 + saline, and SCH23390 + SKF38393 in the ipsi- (i) and contra- (ii) lateral hind paws. **c** Representative images of DRD1 IHC (i) and DRD5 FISH (ii) in the VL-PAG of rats with inserts of positive control stains in the substantia nigra pars reticulata (SNr) for D1R and the paraventricular nucleus of the hypothalamus (PVN) for DRD5 (*n* = 3). **d** Percentage co-localisation of DRD5 FISH probe with main neural cells in naive male Wistar rat VL-PAG (*n* = 3). Representative images (i-iii) of HuC/D showing co-localisation of these markers and DRD5 expression. Small inserts showing individual cell co-localisation with each marker shown at the top right of each image. **e** Percentage co-localisation of DRD5 FISH probe with neuronal subtypes in naive male Wistar rats (*n* = 3). Representative images (i-iii) of VGLUT2 showing co-localisation of these markers and DRD5 expression. Small inserts showing individual cell co-localisation with each marker shown at the top right of each image. White arrowheads indicate positive cells. All data are represented as mean ± SEM. For the development of hypersensitivity (**b**i), using two-way repeated measures ANOVA with a post-hoc Tukey’s multiple comparisons test, * indicates that *P* < 0.05 for contralateral paw responses and ^#^ indicates that *P* < 0.05 for ipsilateral paw responses in comparison to baseline. For mechanical thresholds in response to intra-PAG administration of drug compounds (**b**ii, iii), using Kruskal–Wallis with Dunn’s multiple comparisons tests, * indicates that *P* < 0.05 versus other groups. All scale bars are 100 µm unless stated otherwise.

To discern the effects of replenishing dopamine transmission within the VL-PAG of hemiparkinsonian rats and discover the dopamine receptor subtype involved, we implanted an intracranial cannula into the VL-PAG of Wistar rats after injecting 6-OHDA into the left MFB. Post-mortem analysis confirmed the location of the cannulae within the VL-tier of the PAG (Fig. 3a). As expected, these rats had significant reductions in TH^+^ cells in the ipsilateral SNc (Supplementary Fig. 4) and developed significant reductions in mechanical thresholds in both hind paws at days 7- and 14 post lesion (Fig. 3bi).

After confirming the hypersensitivity, the functional effects of modulating dopaminergic transmission were investigated using the D_1_-like receptor agonist, SKF38393 and the D_1_-like receptor antagonist, SCH23390, alongside their saline controls. Briefly, rats were administered, in randomised order, all four drug combinations directly into the VL-PAG. Following intra-PAG saline-saline treatment or SCH23390-saline treatment, mechanical thresholds were as per day 14 post lesion (Fig. 3bii, iii). In contrast, when given saline-SKF38393 combination, mechanical thresholds in both hind paws were restored to the pre-lesion level (day 0 in 3b(i)), with no noted signs of increased locomotor activity. This increase in mechanical threshold was not observed when the D_1_-like agonist SKF38393 was given after the D_1_-like antagonist SCH23390 (Fig. 3bii, iii). These findings confirm that in a hemiparkinsonian rat, reinstatement of dopamine transmission in the PAG, via D_1_-like dopamine receptor agonist infusion, drives a reversal of the hypersensitivity.

To delineate the subtype of D_1_-like dopamine receptor mediating this antinociceptive action, we performed IHC staining for D_1_R and, in the absence of a suitable antibody, in situ hybridisation for DRD5, the gene coding for D_5_R. While D_1_R was undetected, (Fig. 3ci), DRD5 mRNA was expressed within the VL-PAG of naive rats (Fig. 3cii). DRD5 mRNA was also expressed in the VL-PAG in both the intact and lesioned hemispheres of 6-OHDA MFB-lesioned rats (Supplementary Fig. 4). DRD5 mRNA was exclusively found in HuC/D^+^ neurons and not in GFAP^+^, Iba1^+^, or Olig2^+^ cells (Fig. 3d, Supplementary Fig. 4 and Supplementary Table 1). Further scrutiny revealed that DRD5 was mainly (approx. 80%) expressed in VGLUT2^+^ cells, with a smaller proportion (approx. 15%) expressed in GAD1^+^ cells and the remaining 5% comprised of sparse co-expression in TH^+^ and TPH^+^ cells (Fig. 3e, Supplementary Fig. 4 and Supplementary Table 1).

### Hemiparkinsonian rats have reduced activity in serotonergic cells of the Raphé magnus

To assess the functional activity of hindbrain pain nuclei in unilateral 6-OHDA lesioned rats, we made an intra-plantar injection of capsaicin into the hind paw contralateral to the lesion. This was done to chemically activate primary afferent fibres and by doing so, drive ascending and descending central pain circuitry, and increase the expression the immediate-early response gene, c-FOS, as a proxy for neuronal activity.

**Fig. 4:**
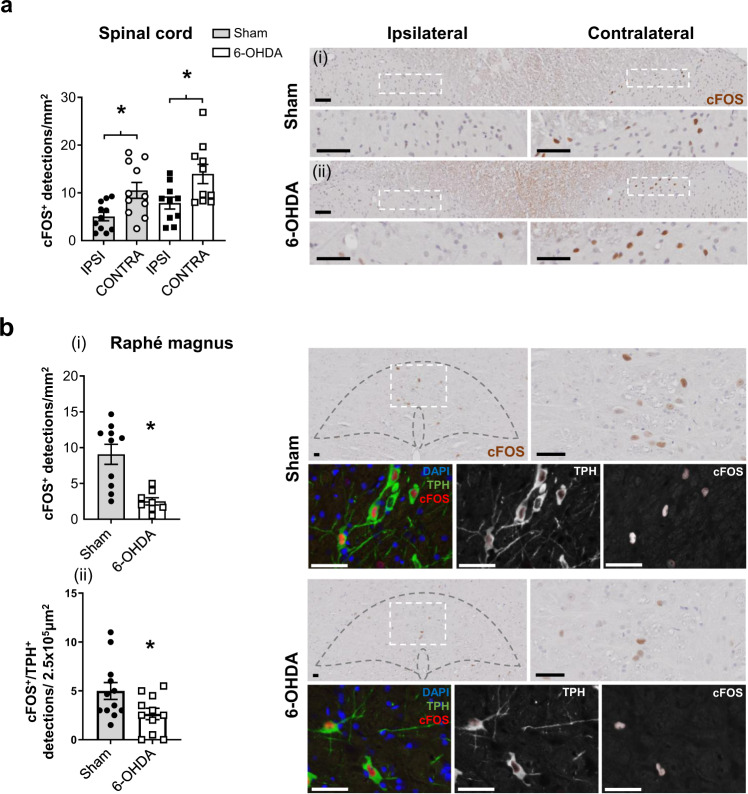
Hemiparkinsonian rats have reduced activity in the Raphé magnus. **a** Number of c-FOS^+^ detections in sham- (*n* = 12) and 6-OHDA (*n* = 11) lesioned rats in the dorsal horn of the spinal cord after being administered intra-plantar capsaicin. Representative images of the sham and 6-OHDA lesioned rat dorsal horns are displayed above magnified images of the dotted box area of ipsi- and contralateral dorsal horns for visual comparison. **b** Number of c-FOS^+^ cell detections (i) and TPH^+^/c-FOS^+^ cell detections (ii) in the RMg with representative figures of sham and 6-OHDA lesioned rats, along with higher magnification images of the dotted box area. All data are represented as mean ± SEM. For histological analyses, Kruskal–Wallis with Dunn’s multiple comparisons tests were performed for spinal cord analysis, and RMg analyses were performed with unpaired *t* tests. * indicates that *P* < 0.05. All images were counterstained using haematoxylin. All scale bars = 50 µm.

Prior effectiveness of the lesions was confirmed by motor deficits and mechanical and thermal nociceptive hypersensitivity compared to sham-lesioned rats (Supplementary Fig. 5). After confirming dopaminergic cell loss in the PAG and SNc of the Parkinsonian animals (Supplementary Fig. 5), histological analysis of the dorsal horn of the lumbar SC revealed an increase in contralateral c-FOS^+^ cells in both sham and 6-OHDA lesioned rats (Fig. 4a). Subsequent investigation in pain processing regions of the brainstem affected in early Parkinson’s disease, identified a significant reduction in c-FOS^+^ detections in the RMg of 6-OHDA lesioned rats compared to sham (Fig. 4bi), with no change in the LC (Supplementary Fig. 5). Co-localisation studies further revealed a significant reduction in c-FOS^+^/TPH^+^ detections in the RMg of 6-OHDA lesioned rats (Fig. 4bii). The proposed reduction in serotonergic neuronal activity in the RMg may underlie the reductions in Met-ENK seen in the SC and the subsequent observed nociceptive hypersensitivity.

### Reduced dopaminergic cells in the PAG and Met-enkephalin levels in the spinal cord in Parkinson’s disease

To establish whether the PAG and SC pathology found in Parkinsonian rats was also present in Parkinson’s disease, we selected PAG and SC tissue from age-matched controls and from Parkinson’s disease cases that exhibited SPPD-like symptoms, and those that did not. No significant difference was present between age at death, post-mortem delay, age at onset of Parkinsonian symptoms or disease duration between all groups (control, SPPD and No SPPD) for PAG or SC samples (Supplementary Fig. 6).

**Fig. 5:**
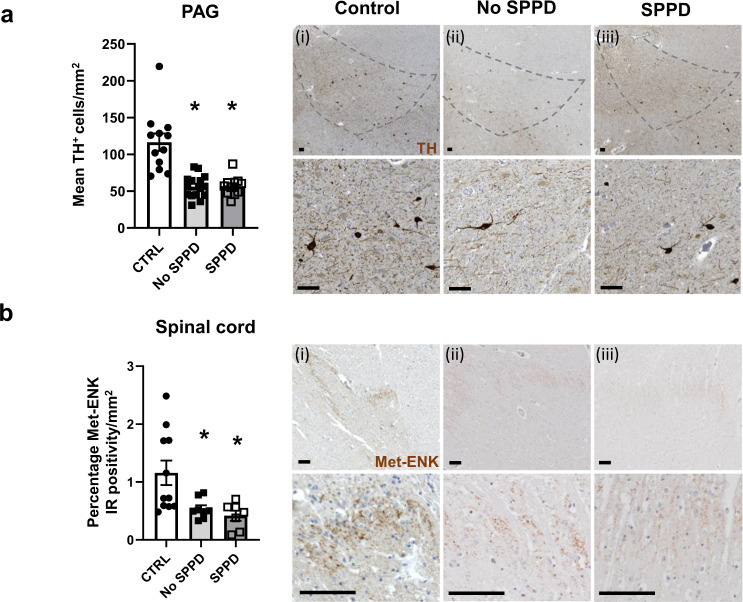
Dopaminergic cell reductions in the PAG and attenuated Met-ENK levels in the spinal cord are seen in Parkinson’s disease. **a** Total number of TH^+^ cells in the VL-PAG of age- and sex-matched controls (*n* = 12) and Parkinson’s disease cases that reported spontaneous Parkinson’s disease-like pain (SPPD; *n* = 11) and those that did not (No SPPD; *n* = 15). Representative images show TH immunostain for Control (i), No SPPD (ii) and SPPD (iii) with a higher magnification image below. **b** Comparison of Met-ENK immunoreactivity in the dorsal horn of control (*n* = 10), Parkinson’s disease with pain (*n* = 7) and without pain (*n* = 9). Representative images for Met-ENK are shown for Control (i), No SPPD (ii) and SPPD (iii) with higher magnification images below. All data are expressed as mean ± SEM. All analysis were performed using Kruskal–Wallis with Dunn’s multiple tests where * indicates that *P* < 0.05 in comparison to control. All scalebars = 100 µm.

Both Parkinson’s disease groups exhibited approximately 50% less TH^+^ cells within the VL-PAG in comparison to control (Fig. 5a). Considering the homogeneity of the SC, and no reports of differential regional expression of Met-ENK in the dorsal horn, we selected one slide per case with 4-5 consecutive sections and averaged the percentage of positive immunostained area for Met-ENK in the dorsal horn of each SC. In control SC, ~1% of the sampled area was positive for Met-ENK. Both Parkinson’s disease groups exhibited a significant >50% reduction in percentage positivity for Met-ENK immunostaining in comparison to age-matched control samples (Fig. 5b). However, there was no significant difference between the SPPD and No SPPD groups for either TH^+^ cell counts in the PAG or Met-ENK staining in the SC (Fig. 5a, b).

### Microgliosis and α-synuclein oligomers in the spinal cord of Parkinson’s disease cases with pain

**Fig. 6:**
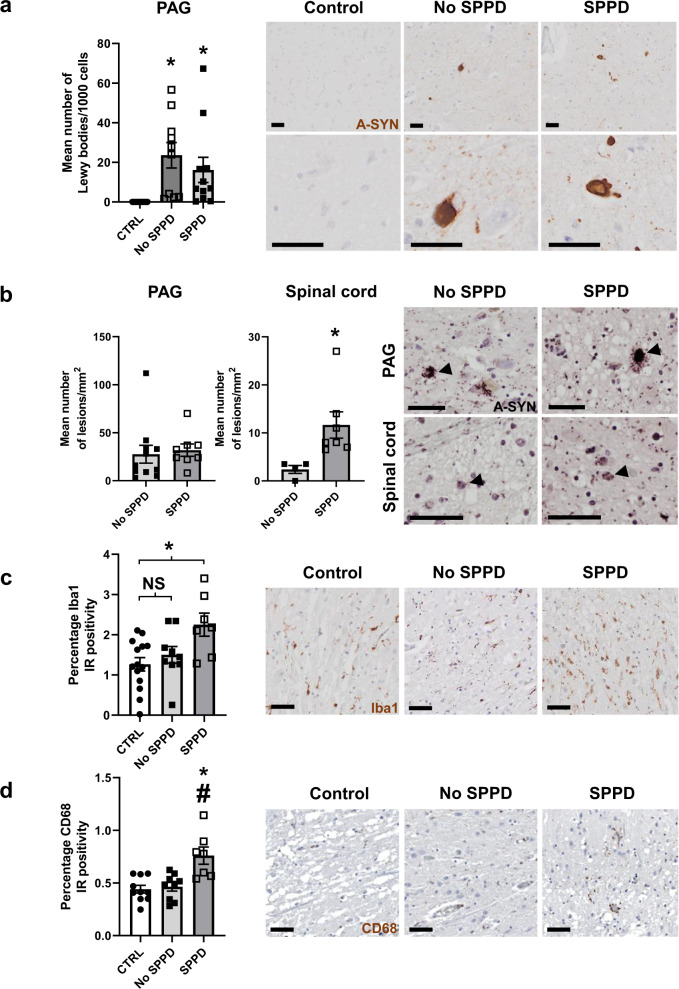
Microgliosis in Parkinson’s disease cases exhibiting pain is linked with α-synuclein oligomers in the dorsal horn of the spinal cord. **a** Mean number of Lewy bodies per 1000 haematoxylin-positive nuclei in 1 mm^2^ in control (*n* = 12), Parkinson’s disease cases with (*n* = 11) and without (*n* = 15) reported SPPD-like pain, alongside representative images of α-synuclein immunostain for each group. **b** Comparison of α-synuclein oligomer positivity cells in the PAG and spinal cord in Parkinson’s disease patients with no SPPD-like pain (PAG; *n* = 8, spinal cord; *n* = 4) and those with SPPD-like pain (PAG; *n* = 11, spinal cord; *n* = 7). Oligomer lesion-positive cells are indicated by black arrows in the representative images. **c** Iba1 immunoreactivity in the dorsal horn of spinal cord of control (*n* = 14), Parkinson’s disease with (*n* = 7) and without (*n* = 9) SPPD-like pain, alongside representative images. **d** CD68 immunoreactivity in the dorsal horn of spinal cord of control (*n* = 10), Parkinson’s disease with (*n* = 7) and without (*n* = 9) SPPD-like pain, alongside representative images. All data are expressed as mean ± SEM. All analyses were performed using Kruskal–Wallis with Dunn’s multiple tests. * indicates that *P* < 0.05 in comparison to control, ^#^ indicates that *P* < 0.05 when comparing SPPD and no SPPD groups, and NS = not significant. All scale bars = 50 µm.

To establish whether the PAG-dopaminergic or spinal-enkephalinergic pathology coexisted with Lewy pathology, we performed immunostaining for α-synuclein in the SC and PAG. While some Parkinson’s disease cases did not have any Lewy pathology within the VL-PAG, overall, both Parkinson’s disease groups displayed significant increases in Lewy bodies when compared to control, but no difference in Lewy body burden between SPPD and No SPPD groups (Fig. 6a). In SC samples, despite two cases having Lewy pathology in the ventral laminae of the SC, no samples from any group exhibited Lewy pathology within the dorsal horn (Supplementary Fig. 5). We subsequently investigated the number of α-synuclein oligomeric lesions in the PAG and SC of the Parkinson’s disease cases. This revealed no difference in lesion numbers in the PAG between Parkinson’s disease cases, but the SPPD group displayed significantly more pre-aggregate lesions in the dorsal horn of the SC than the No SPPD group (Fig. 6b). Further investigation showed no difference in astrocytosis between any of the groups (Supplementary Fig. 6) but the SPPD samples exhibited elevated Iba1 levels in the dorsal horn when compared with controls (Fig. 6c), and increased CD68, when compared to both the control and No SPPD groups (Fig. 6d).

Overall, the dopaminergic and Met-ENK changes seen in the PAG and SC, respectively, of hemiparkinsonian rats with hyperalgesia matches findings in post-mortem samples from Parkinson’s disease patients, in which reduced sensory thresholds are known to occur. However, these changes do not appear to be linked to the manifestation of SPPD which may instead be explained by the inflammatory response, possibly due to an increase in α-synuclein oligomer formation, in the dorsal horn of the SC in a subset of Parkinson’s disease patients.

## Discussion

Our studies into the potential mechanisms by which pain develops in Parkinson’s disease have identified that reductions in dopaminergic neurons in the VL-PAG and Met-ENK reductions in the SC occur contemporaneously in the MFB and VL-PAG 6-OHDA lesioned rats that display mechanical and thermal hypersensitivity. Pharmacological activation of D_1_-like receptors in the VL-PAG, identified as a DRD5^+^ phenotype located on glutamatergic neurons, alleviates the hypersensitivity seen in the Parkinsonian model. In addition, the Parkinsonian state leads to reduced activity of serotonergic neurons in the RMg of 6-OHDA lesioned rats. The PAG-dopaminergic and spinal Met-ENK reductions were also seen in post-mortem samples from Parkinson’s disease cases, in which reduced sensory thresholds are known to occur in life, but these do not differentiate the SPPD and No SPPD groups. However, patients exhibiting SPPD exhibited microgliosis in the dorsal horn of the SC where there was increased α-synuclein oligomer formation. Thus, two independent mechanisms may be involved in driving the nociceptive hypersensitivity and SPPD.

The unilateral 6-OHDA-lesioned rat model has previously been shown to present with bilateral hypersensitivity from day 7 post lesion onwards, as seen with multiple nociceptive modalities^2^. We successfully reproduced this phenotype, which replicates the reduced pain thresholds seen in Parkinson’s disease^11,14,15,41^. The pathological changes we found in this hemiparkinsonian model provide insight into what changes may drive the observed hypersensitivity.

Within the SC of hemiparkinsonian rats, and Parkinson’s disease samples, we identified reductions in Met-ENK in the dorsal horn, which refined the previous report of overall reductions in Met-ENK from hemisected SC homogenates^35^. The main afferents that control spinal Met-ENK levels originate from the brainstem, namely the LC and RMg, in which we found no changes in DBH^+^ and TPH^+^ cells, respectively. Our findings in the RMg are at odds with those of Wang et al.^40^, who reported a significant reduction in serotonergic neurons in the RMg. However, their study used a bilateral SNc lesion model which is a more severe model than the unilateral MFB model used here. Regardless, we did find a significant reduction in activity of these neurons, deduced via reduced c-FOS staining in the TPH^+^ cells of the RMg in Parkinsonian rats.

We conclude that Parkinsonian neuropathology in the rodent leads to a dysfunction in spinal-inhibitory signals, without overt brainstem serotonergic or noradrenergic pathology. However, the fold change in c-FOS^+^ detections verses that of the c-FOS^+^/TPH^+^ neurons indicate that further functional changes in non-serotonergic neuronal populations within the RVM may play a role in the nociceptive hypersensitivity in this model^42^.

Though many groups have deduced that the descending pain modulatory pathway is a pivotal factor driving the hypersensitivity in preclinical models of Parkinson’s disease^35,40^, no studies have established the anatomical or functional connection between nigrostriatal degeneration and the pain phenotype. When 6-OHDA is injected into the MFB, as in our studies, it is taken up and transported anterogradely and retrogradely along the dopaminergic cells that project through it^43^. Though the main target for this Parkinsonian model is the nigrostriatal fibres, the mesolimbic axonal projections from dopaminergic cells in the VTA are also affected^44^. The 50% cell loss we revealed in the VL-PAG cannot be caused downstream of dopaminergic cell death in the nigrostriatal tract since tracing studies show no dopaminergic connections between these two brain areas^45,46^. However, given the connection between rostral portion of the PAG and the VTA^45,46^, we propose that the PAG-dopaminergic cell loss following MFB injections of 6-OHDA is mediated via the VTA afferent fibres which project through the MFB to the nucleus accumbens^47,48^. We further noted that, even with the direct intra-PAG 6-OHDA injection, there still remained ~50% of the dopaminergic cell population. One reason for this may relate to the abundance of calretinin-positive neurons in the VL-PAG^49^, which have been shown to be resistant to 6-OHDA lesioning in rats^50^.

The PAG has been implicated in driving pain in other neurological conditions in humans, such as in multiple sclerosis and mild traumatic brain injury^51,52^. In Parkinson’s disease, a recent fMRI study reported reduced activity in the PAG which was linked to increased pain sensation using thermal QST^27^, an index of hypersensitivity that back-translates well to the hypersensitivity noted in the 6-OHDA lesioned rat model of Parkinson’s disease. Although these fMRI studies offered no insight into what neurons were involved, investigating only global reductions in PAG activity, our findings suggest this reduced activity may reflect, in part, a reduction in dopaminergic tone in the VL-PAG. The antinociceptive role of dopamine in the PAG is well-established. Our findings add to this in two important ways. Firstly, we show that selective ablation of dopamine cells in the VL-PAG alone is sufficient to drive a hypersensitive phenotype in naive rats, revealing a direct relationship between the loss of these cells and the hypersensitivity. Secondly, we show that not only do dopamine reductions in the PAG of hemiparkinsonian rats accompany nociceptive hypersensitivity, but that intra-PAG-dopaminergic stimulation of the D_1_-like receptor reverses this hypersensitivity. We further identified that the D_1_-like receptor involved was likely the D_5_R subtype. Though no studies have investigated D_5_R expression in the PAG of humans, human whole-brain transcriptome databases have confirmed that DRD5, and not DRD1, is preferentially enriched in the lateral and ventral tiers of the PAG^53,49^. We further revealed that DRD5 is mainly expressed in glutamatergic neurons in the VL-PAG establishing a pain-related link between dopaminergic and glutamatergic signalling pathways within the PAG. Opto- and chemo-genetic studies have corroborated this by showing that VL-PAG glutamatergic and dopaminergic neurons similarly control nociceptive signals bidirectionally by inhibiting pain when stimulated and vice versa^54,55^.

Similarly, Bennaroch et al.^56^ have seen dopaminergic pathology in the PAG of two other synucleinopathies, multiple system atrophy (MSA) and dementia with Lewy bodies. Though their study associated the reduction in dopaminergic neurons in the PAG with fatigue and altered circadian rhythm^57^, our findings suggest a further link with gating pain signals via regulation of spinal Met-ENK levels. Given MSA is reported to have a manifestation of pain comparable to that seen in Parkinson’s disease^58^, it is feasible that the shared MSA and Parkinson’s disease PAG pathology contributes to this. If so, pain in both these conditions may benefit from similar therapeutics that counter the reduced transmission in the dopamine-driven PAG-RMg-SC pathway. This hypothesis is reinforced by the efficacy of the SNRI, duloxetine, which has been shown to reduce reports of central pain in Parkinson’s disease^59^, as well as being antinociceptive in the 6-OHDA lesioned rat model of Parkinson’s disease^35^. Though duloxetine fails to reverse QST thresholds in people with Parkinson’s disease^59^, its moderate success suggests that central monoaminergic manipulation may be the way forward.

According to our findings in PAG and SC samples from Parkinson’s disease cases, the respective reductions in dopaminergic neurons and Met-ENK, associated with hypersensitivity in the rodent model, are independent from the manifestation of SPPD. A review of the QST studies performed in people with Parkinson’s disease revealed that pain thresholds are reduced when compared to healthy age-matched controls, regardless of whether the patients report spontaneous pain or not^2^. Accordingly, pain thresholds have been broadly reported to exhibit no significant difference between Parkinson’s disease patients that report SPPD or not^2^. Given our findings linking dopaminergic cells in the PAG and Met-ENK levels in the SC to hypersensitivity, the pathology we have identified could be the mechanism by which hypersensitivity in Parkinson’s disease, rather than SPPD, develops. This has been reflected in previous studies looking into reduced function of the PAG in early stages of Parkinson’s disease and linking it to increased pain perception^27^, before SPPD develops.

The question then remains, what lies behind the manifestation of SPPD. We have identified two key components that may contribute: an increase in activated microglia in the dorsal horn, coupled with an increase in neurons affected by pre-aggregate α-synuclein oligomer deposition, in the SPPD group compared to those that did not exhibit SPPD. Microglia have been strongly implicated in playing a pivotal role in neurodegenerative diseases^60^, including Parkinson’s disease^61^, with emphasis on their contribution to the pathogenesis via exacerbated proinflammatory response in the presence of α-synuclein oligomers and aggregates^62–64^. Despite studies showing that the SC is impacted in Parkinson’s disease by the presence of Lewy pathology^32^, no studies have investigated the link between this pathology in the dorsal horn of the SC and the manifestation of pain. That said, in the pain field in general, numerous studies have shown that microgliosis contributes to facilitation of nociceptive signals^65^. Preclinically, several peripheral neuropathy models in rats present with significant increases in microglia in the dorsal horn of the SC as well as exhibiting nociceptive hypersensitivity^66,67^. In league with our own findings, a study by Garcia et al.^68^ showed that intra-striatal injection of preformed fibrils of α-synuclein in mice trigger degeneration and microgliosis in brain regions not only with, but also in the absence of, α-synuclein inclusions. This indicates that oligomers are the predominant driver of neurodegeneration in early stages of Parkinson’s disease and, in line with our findings, may initiate inflammatory responses in key pain-related regions ahead of any manifestation of SPPD.

In summary, our findings show that dopaminergic cell loss in the VL-PAG is an important pathological characteristic of Parkinson’s disease that may drive hypersensitivity during the early stages of the condition. In combination with the reductions in Met-ENK in the SC, this may lead to a sub-clinical manifestation of hypersensitivity that ultimately leads to long term changes in plasticity in pain circuitry. This may be further compounded by a neuroinflammatory component that is linked to oligomer formation within the SC, which we propose may result in the reports of SPPD in some patients. In this respect, we show here that hypersensitivity and SPPD may have differing aetiologies in Parkinson’s disease. Our findings highlight numerous therapeutic strategies that may offer analgesic relief for people with Parkinson’s disease, including D_5_R agonists and serotonergic reuptake inhibitors for treating the hypersensitivity and anti-inflammatory agents for treating SPPD.

## Methods

### Animals

All animal procedures adhered to the ARRIVE guidelines, were in accordance with the UK Animals (Scientific Procedures) Act, 1986 and EU Directive 2010/63/EU, and were approved by King’s College London Animal Welfare and Ethical Review Body. Further approval of individual study designs was provided by the Interim Director of the King’s College London Biological Services Unit. Experiments were performed on 250 g adult male Wistar rats (Envigo) with a total of 83 rats used and were randomly assigned groups via random number generator with sample sizes calculated using G*Power^69^. Male animals were used for these studies to exclude the effect of the oestrogen cycle which is known to impact nociceptive thresholds. Animals were housed in the Biological Services Unit, King’s College London, maintained on 12-h day / night cycle with ad libitum access to food and water, and were acclimatised for 7 days prior to experiments. ‘Lesioned’ rats with no dopaminergic cell loss in the SNc, and those with displaced intra-PAG cannulae were excluded from analyses. The experimenter was blinded for all surgical, behavioural, and histological procedures and analyses. Procedures were performed under personal licence I6D889531 and project licence PB944CCE3.

### Unilateral 6-hydroxydopamine lesioning and intra-periaqueductal grey (PAG) infusion of drugs

At day 0, rats were pre-treated (i.p.) with 5 mg/kg pargyline and 25 mg/kg desipramine. Thirty minutes later, under isofluorane anaesthesia (5% induction, 2–3% maintenance), they were infused (0.5 μl/min) with either 12.5 μg 6-OHDA.HCl (Tocris Bioscience, UK) in 2.5 μl saline containing 0.2% ascorbic acid or vehicle (2.5 μl saline containing 0.2 % ascorbic acid) into the left medial forebrain bundle (*n* = 12, sham; *n* = 12, 6-OHDA) (MFB; AP, –2.6 mm; ML, + 2.0 mm; DV, –8.8 mm from bregma) or the left VL-PAG (*n* = 12, sham; *n* = 12, 6-OHDA) (AP, –7.2 mm; ML, + 0.8 mm; DV, –6.0 mm from bregma), as defined by Paxinos and Watson^70^. For the intra-PAG-dopaminergic drug administration study, a separate group of MFB-lesioned rats (*n* = 5) was additionally implanted with a 2.5 cm single-barrelled 23 G stainless steel cannula, 1 mm above the left VL-PAG (AP, –7.2 mm; ML, + 0.8 mm; DV, –5.0 mm, from bregma). The cannula was fixed in place using cyanoacrylate gel, a protective headcap fixed to the skull surface and a stainless steel 30 G stylet placed in the cannula to prevent blockages. Two weeks post surgery, after mechanical hypersensitivity was confirmed, the cannulated rats were lightly restrained and received 0.5 µl intra-PAG injection of each drug combination via a 30 G needle. The first drug (either 0.5 µl of saline or the D_1_-like receptor antagonist, SCH23390.HCl; Tocris Biosciences, UK; 32 mM) was administered 30-min before the second drug (either 0.5 µl saline or the D_1_-like receptor agonist, SKF38393.HBr; Tocris Biosciences, UK; 6 mM). Rats were left to acclimatise for 15 min post-injection before nociceptive behavioural assessments commenced. All rats received each drug combination through randomised Latin-square with a two-day washout period between each testing day.

### Motor and nociceptive assessment

Cylinder test assessment of forelimb akinesia was performed in all 6-OHDA MFB injection studies 1-day prior to surgery and on day 14 post lesion, as previously described^71^. Similarly, nociceptive tests assessing mechanical (von Frey; VF), cold (acetone/dry ice) and heat (Hargreaves) thresholds were assessed prior to 6-OHDA surgery and on day 7 and 14 post lesion, except for the initial study where cold and mechanical thresholds were assessed on days 3, 7, 14, 21 and 28 post lesion. All nociceptive tests were repeated three times for each hind paw.

For VF testing, rats were placed in transparent acrylic boxes with wire mesh floors. For manual testing, VF monofilaments (Stoelting) were firmly applied to the plantar surface of each hind paw for 5 s. The up-down method of Dixon^72^ was used to estimate the 50% withdrawal threshold (g). For automated testing, the Dynamic plantar aesthesiometer (Ugo Basile, Italy) was used to assess static mechanical withdrawal thresholds. This automated VF was set to apply a force of 0–50 g with a ramp increase of 2.5 g/s for a maximum of 20 s.

Heat thresholds were assessed by using the Hargreaves apparatus (Ugo Basile, Italy). Rats were placed in a 20-cm square Perspex box atop a translucent, glass screen elevated at 50 cm. The generator emitted an infrared beam to heat the hind paw and the withdrawal latency was calculated with a cut-off of 20 s.

Cold thresholds in the timeline study were assessed by the acetone test. Rats were placed in the VF chambers. 0.1 ml of acetone was applied to the plantar region of the hind paw using a 1 ml syringe barrel and the number of flicks and licks was measured after application for 1 min.

As described in ref. ^73^, the dry-ice test was performed in the same apparatus as the Hargreaves test. A truncated 2.5-ml syringe barrel filled with dry-ice powder was applied to the surface of the glass screen below the hind paw, and the time taken for the rat to withdraw its paw was measured with a cut-off of 20 s.

### Induction of c-FOS in the CNS

To measure functional activity in hindbrain pain nuclei, sham and 6-OHDA lesioned rats (*n* = 12 per group) were prepared and tested as described above. Two weeks post lesion, rats received 1.6 g/kg (i.p.) urethane anaesthesia 30 min prior to intra-plantar injection of 30 µg of capsaicin (Tocris Bioscience, UK) in 50 µl of vehicle (15% ethanol v/v in Milli-Q water). Two hours after capsaicin administration, animals were processed for tissue preparation, exactly as outlined below.

### Tissue preparation

Upon completion of the above in vivo protocols, rats were terminally anaesthetised using i.p. phenobarbital overdose then perfused with 1× PBS solution and 10% neutral buffered formalin. Brains and spinal cords (SC) were dissected for all 6-OHDA lesion studies. For the longitudinal study, the dorsal root ganglia (DRG) at the level of L3, L4 and L5 were also dissected, however, only half of the rats were randomly selected for post-mortem analysis in this study, as the remaining animals were used for further behavioural investigations for an unrelated study (data not shown). The samples were then paraffin wax- embedded and tissue blocks cut into 7-μm thick sections using a microtome (Leica RM2235, Leica Biosystems, UK) and mounted onto Superfrost Plus slides.

### Post-mortem human case samples

Brain and SC samples were obtained from the Queen Square Brain Bank for Neurological Disorders (UCL, Institute of Neurology) and the Parkinson’s disease UK Brain Bank (Imperial College London). All samples were donated with full informed consent. Accompanying clinical and demographic data of all cases were stored electronically in compliance with the 1998 data protection act (Supplementary Table 2). Ethical approval for the study was obtained from the NHS research ethics committee, UK and in accordance with the human tissue authority’s code of practice and standards under licence number 12521. Parkinson’s disease cases were stratified into groups that exhibited pain and those that did not, using clinical notes provided by the respective Brain Banks. Symptoms included pain, burning, and tingling sensations experienced in the legs, back, shoulder, and chest. The exclusion criteria for Parkinson’s disease patients that exhibited pain were evidence of angina, urinary tract infection, dyspepsia, active chemotherapy, diabetes, adverse drug reactions, fever/infections, dysuria, pre-existing injury, and rheumatoid arthritis.

### Histological and molecular biological methods

#### Immunohistochemistry

For immunohistochemistry (IHC), slide-mounted sections were dried at 60 °C overnight then deparaffinised in xylene, rehydrated in decreasing grades of ethanol (100, 90, 70%) and incubated in H_2_O_2_ (0.3%) solution for 10 min. For heat-induced antigen retrieval, slides were transferred to an appropriate boiling solution of either 0.1 M TRIS-EDTA buffer (pH 9.0) or 0.1 M citrate buffer (pH 6.4) (Supplementary Table 3). Immunostains for α-synuclein required a further 15 min pre-treatment in 98% formic acid before blocking with 1% BSA in TBS solution at RT for 10 min. All sections were incubated for 2 h with primary antibody at room temperature (RT) then for 1 h with appropriate biotinylated IgG secondary antibody (1:200) or fluorescent secondary (1:1000). Details of all antibodies used in the study are given in Supplementary Table 3, and their validation details noted in Supplementary antibody data file. For chromogenic staining, slides were then incubated in pre-conjugated Strept(avidin)–Biotin-Complex (ABC; Vector Laboratories), then submerged in 3,3ʹ-Diaminobenzidine chromogen solution and where applicable, sections were counterstained in Mayer’s haematoxylin. Finally, slides were dehydrated in increasing grades of ethanol (70, 90, and 100%), cleared in xylene, and mounted using DPX. For fluorescent staining, post-secondary incubation, the sections were incubated in DAPI and mounted using Vectashield anti-fade mounting medium (Vector Laboratories).

#### Fluorescent in situ hybridisation

PAG sections from the brains of naive (*n* = 3) and 6-OHDA MFB-lesioned (*n* = 3) rats, prepared as described above, were used for FISH. The RNAscope™ protocol was performed as detailed by the user manual provided by ACD Bio-techne for the RNAscope™ Multiplex Fluorescent Reagent Kit V2 Assay. After deparaffinising, dehydrating, pre-treatment and antigen retrieval, each section was treated with RNAscope™ Protease Plus for 30 min at 40 °C. The C1-DRD5 (DRD5, accession no. NM_012768.1), and where applicable the C2-GAD1 (GAD1, accession no. NM_0.17007.2), C3-VGLUT1 (Slc17a7, NM_053859.4), and C3-VGLUT2 (Slc17a6, NM_053427.1) probes were added to the section using dilution factors recommended by the manufacturers and were incubated for 2 h at 40 °C. After a series of amplification steps, slides were treated with their respective horseradish peroxidase solution followed by incubation with the appropriate Opal™ dye (1:1000), followed by the horseradish peroxidase blocker for 30 min and 15 min, respectively, at 40 °C. For combined immunofluorescence and in situ hybridisation the slides were finally stained with the same immunofluorescence protocol mentioned above.

#### Proximity ligation assay

This proximity ligation assay (PLA) protocol adhered to the steps laid out by Roberts et al.^74^ and the DuoLink® manufacturer’s instructions. Prior to staining, the PLA probes were conjugated with the α-synuclein antibodies using the Duolink® PLA Probemaker kit. For the staining, tissue was pre-treated as for IHC. After antigen retrieval tissue was covered by blocking solution provided in the Duolink® In situ Detection Reagents Brightfield kit for 1 h at 37 °C. The previously formulated probes were diluted in the PLA probe diluent (1:100 for PLUS and MINUS), added to the sections and incubated overnight at 4 °C. The slides were then washed 4 × 5 min with Duolink® washing buffer. The enzymatic ligation step was performed for 1 h at 37 °C then sections were incubated at RT in the amplification solution followed by the detection solution for 2.5 h and 1 h, respectively. The sections were finally incubated in substrate solution for 20 min at RT, counterstained then dehydrated, cleared with xylene, and mounted with coverslips using DPX.

### Histological quantification

#### Single chromogen analyses

Tyrosine hydroxylase (TH^+^) cell loss was analysed in the SNc and the PAG across three rostro-caudal levels: for SNc; −5.0, –5.5 and −6.0 mm from bregma and for PAG; −7.08, −7.56, −8.04 mm from bregma^70^. After manual cell counting, the data from 3 to 4 consecutive sections from each area was averaged for each animal. For other CNS areas, a similar procedure was performed across one level, apart from the SC, which adhered to the technique performed by Aman et al.^75^. c-FOS^+^ cells were quantified using QuPath. Areas of the RMg, locus coeruleus (LC), and dorsal horn of the SC ( ≥1mm^2^) were annotated and analysed using the positive cell detection tool with the intensity threshold parameters defined by the mean nucleus 3,3ʹ-diaminobenzidine optical density and a readout of number of positive detections per mm^2^. For the human tissue analyses, PAG samples were obtained from within +34.5 mm to +39 mm from the obex, at the level of the superior colliculus^76^, and SC was taken from lower cervical and upper thoracic areas. For the PAG, eight sections 200 μm apart were obtained per case and stained for TH as described previously. Once imaged and using QuPath, an ROI of 1 mm^2^ was annotated in the VL-PAG^76^. The number of TH^+^ cells were counted manually and summed throughout the eight sections for each case. VL-PAG α-synuclein and all SC staining was performed on one representative slide per case, with a minimum of four sections per slide for the SC. To quantify Lewy body burden, the number of Lewy bodies was counted manually, as per TH cell count and normalised against the number of positive nuclei (per 1000) detected via the cell detection script on QuPath whilst glial fibrillary acidic protein (GFAP), ionised calcium-binding adaptor molecule 1 (Iba1), cluster of differentiation 68 (CD68), and Met-ENK analyses in the SC utilised the thresholder tool in QuPath. As for the analysis of PLA stains, positive identification of a α-synuclein PLA lesion was in accordance with the criteria outlined by Roberts et al.^74^ within ≥1 mm^2^.

#### Co-localisation analyses

For the co-localisation analyses, four random 500 µm^2^ areas were selected within the medial VL-PAG of naive rats. The number of DRD5^+^ neurons, the number of cells expressing the second FISH/IHC marker, and the number of DRD5^+^ cells expressing the second marker were counted using the colour deconvolution tool and channel viewer in QuPath. The percentage of DRD5^+^ cells that were double labelled was then determined. The same technique was adopted to assess tryptophan hydroxylase (TPH) and c-FOS co-localisation in the RMg, again using a sampling area of 500 µm^2^.

#### Digital image acquisition and statistical analyses

All fluorescent and monochromogenic-stained tissues were imaged using the Apotome with Aksiovision V2 software. However, for representative images and tissues that were 3,3ʹ-diaminobenzidine and haematoxylin stained, Axioscan 7 and Olympus VS120 slide scanners were used. Images were analysed using ImageJ and QuPath^77^. GraphPad Prism 9 was used for data presentation and statistical analyses for all work performed. The details of specific statistical tests used were stated in figure legends. All figures were made using a combination of Microsoft PowerPoint and BioRender.

### Reporting summary

Further information on research design is available in the Nature Research Reporting Summary linked to this article.

## Supplementary information

Supplementary Files for Buhidma, Hobbs, Malcangio and Duty

Reporting Summary

## Data Availability

According to UK research councils’ Common Principles on Data Policy, all data supporting this study are contained within the paper or Supplementary Files. All raw data for all analyses (e.g., IHC/FISH) as well as further additional information will be openly available upon request from the corresponding author.
