# Supplementary material for: Periaqueductal grey and spinal cord pathology contribute to pain in Parkinson’s disease
Source: NPJ Parkinsons Dis. 2023 Apr 26;9:69. doi: 10.1038/s41531-023-00510-3 (PMC10133233; doi:10.1038/s41531-023-00510-3)
Supplement: Supplementary file 1 — Supplementary Files for Buhidma, Hobbs, Malcangio and Duty [file 41531_2023_510_MOESM1_ESM.pdf]

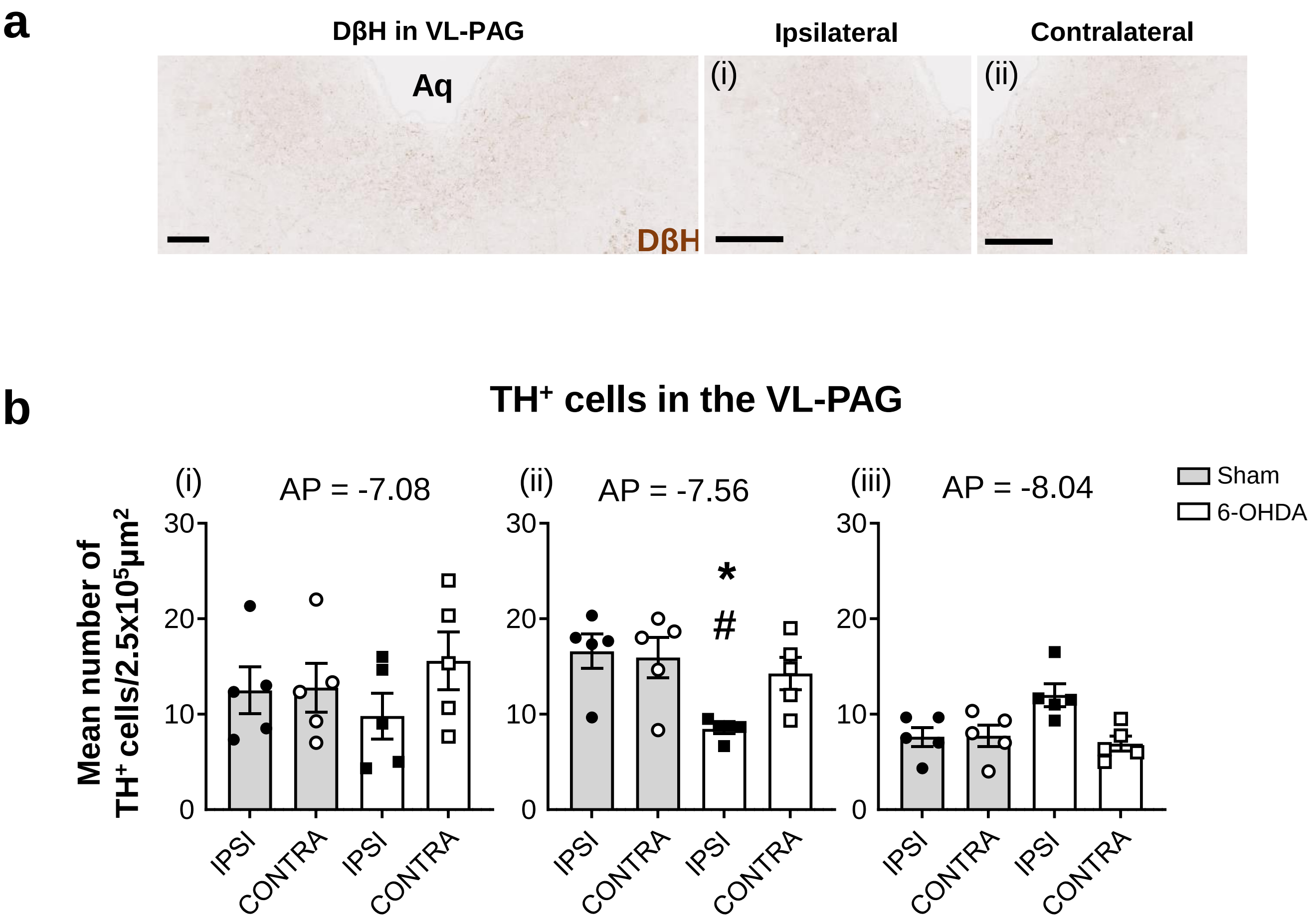

Supplementary Figure 1: Selective dopaminergic pathology is seen in different regions of the ventrolateral periaqueductal grey (VL-PAG). (**a**) Representative immunohistochemical staining showing no dopamine β-hydroxylase (DβH) positive cells in either the (i) ipsi- or (ii) contra-lateral ventrolateral periaqueductal grey (VL-PAG) of a sham rat, supporting tyrosine hydroxylase (TH^+) staining in this region as a selective marker for dopaminergic cells. (**b**) Mean number of TH^+ cells in the ipsi- and contra-lateral VL-PAG across rostral (i), medial (ii), and caudal (iii) areas in sham- (*n* = 6) and 6-hydroxydopamine (6-OHDA)- (*n* = 5) lesioned rats. All data are mean ± S.E.M. Using Kruskal-Wallis with Dunn's multiple comparisons tests, * indicates $P<0.05$ comparing sham with 6-OHDA and # indicates $P<0.05$ for 6-OHDA ipsi- versus contra-lateral sides of the VL-PAG. All scalebars = 200 µm. Aq = Sylvian Aqueduct.

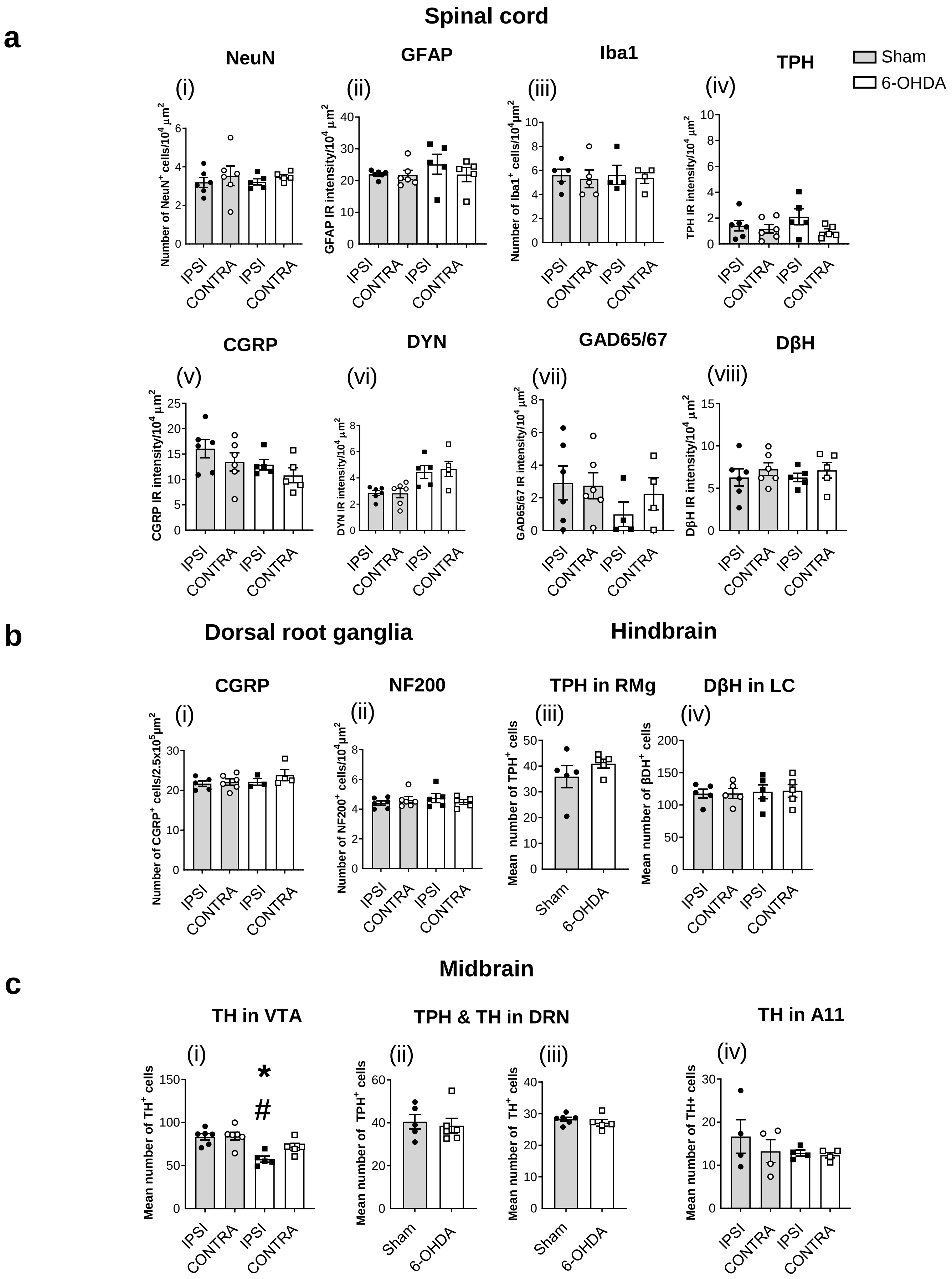

a
Spinal cord
NeuN
GFAP
Iba1
TPH
Sham
6-OHDA
(i)
(ii)
(iii)
(iv)
Number of NeuN+ cells/10^4 µm^2
GFAP IR intensity/10^4 µm^2
Number of Iba1+ cells/10^4 µm^2
TPH IR intensity/10^4 µm^2
IPSI
CONTRA
IPSI
CONTRA
CGRP
DYN
GAD65/67
DβH
(v)
(vi)
(vii)
(viii)
CGRP IR intensity/10^4 µm^2
DYN IR intensity/10^4 µm^2
GAD65/67 IR intensity/10^4 µm^2
DβH IR intensity/10^4 µm^2
b
Dorsal root ganglia
Hindbrain
CGRP
NF200
TPH in RMg
DβH in LC
(i)
(ii)
(iii)
(iv)
Number of CGRP+ cells/2.5x10^5 µm^2
Number of NF200+ cells/10^4 µm^2
Mean number of TPH+ cells
Mean number of βDH+ cells
Sham
6-OHDA
c
Midbrain
TH in VTA
TPH & TH in DRN
TH in A11
(i)
(ii)
(iii)
(iv)
*
#
Mean number of TH+ cells
Mean number of TPH+ cells
Mean number of TH+ cells
Mean number of TH+ cells

Supplementary Figure 2: Lack of pathology seen in pain related regions of the CNS and PNS of hemiparkinsonian rats. (**a**) Analyses of laminae I-III of the dorsal horn in the spinal cord (i-viii) showing the mean number of $NeuN^+$ cells (i), GFAP intensity (ii), $Iba1^+$ cell counts (iii) and immunoreactivity intensity of TPH (iv), CGRP (v), dynorphin (vi), GAD65/67 (vii), and DβH (viii). (**b**) Analyses of DRGs showing intensity of CGRP (i) and NF200 (ii) per 100 μm^2 and analyses of brainstem nuclei RMg and LC for TPH^+ and $D\beta H^+$ cell numbers, respectively. (**c**) Analyses of midbrain nuclei showing the mean number of TH^+ cells in the VTA (i), number of TPH^+ (ii) and TH^+ (iii) cells in the DRN, and TH^+ cells in the A11 nucleus of the hypothalamus (iv). All data are mean ± S.E.M. Using Kruskal-Wallis with Dunn's multiple comparisons tests, * indicates $P<0.05$ comparing sham with 6-OHDA and # indicates $P<0.05$ for 6-OHDA ipsi- vs. contra-latera sides of the brain. Mann-Whitney test was used to analyse medial nuclei that contain one nucleus in the brain, such as DRN and the RMg.

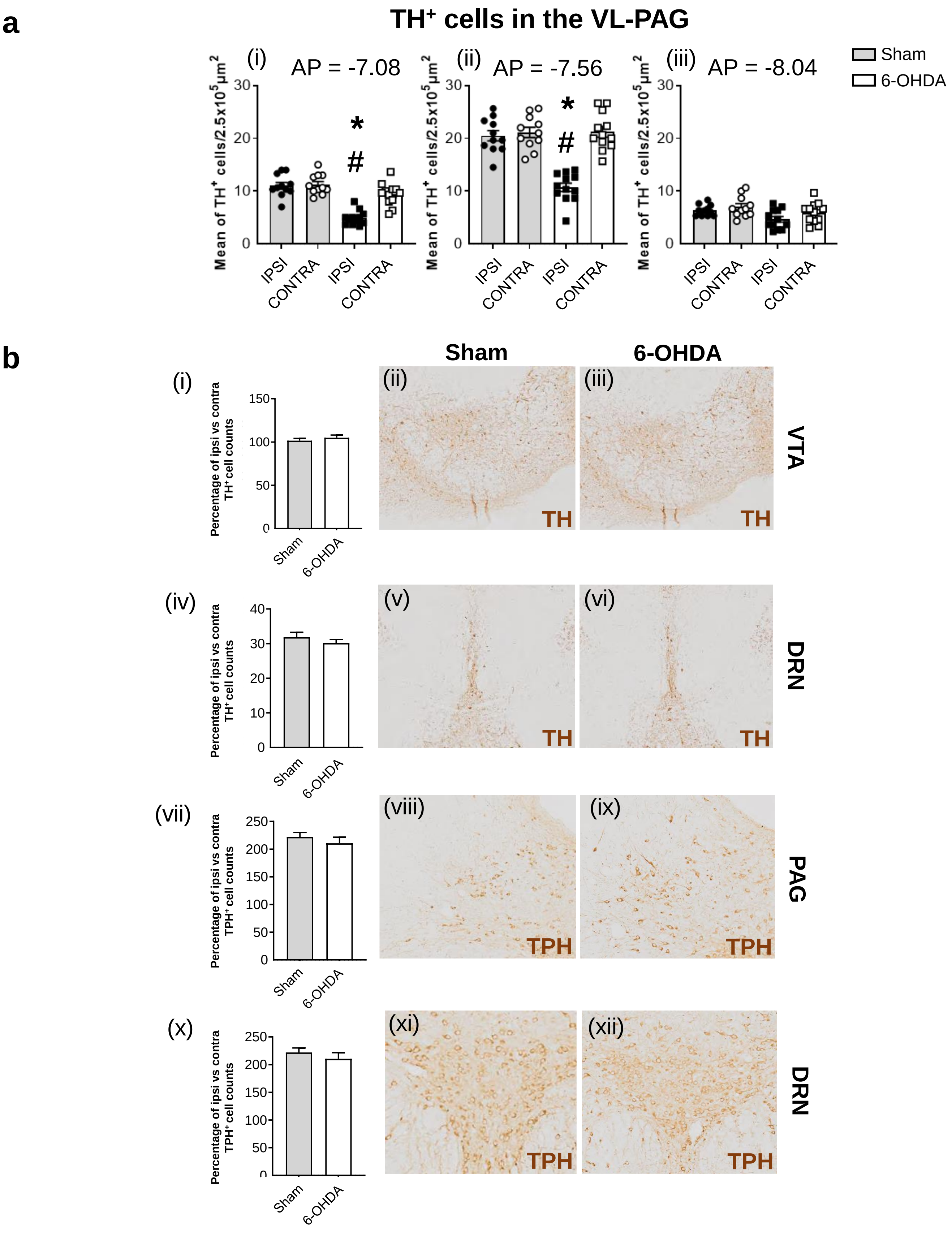
a
TH+ cells in the VL-PAG
(i) AP = -7.08
(ii) AP = -7.56
(iii) AP = -8.04
Sham
6-OHDA
Mean of TH+ cells/2.5x10^5µm^2
IPSI
CONTRA
b
Sham
6-OHDA
Percentage of ipsi vs contra TH+ cell counts
Percentage of ipsi vs contra TPH+ cell counts
VTA
DRN
PAG
TH
TPH

Supplementary Figure 3: Pathology in midbrain of PAG-6-OHDA-lesioned rats is restricted to the dopaminergic cells of the VL-PAG. (**a**) Mean number of TH^+ cells in 3 different areas of the VL-PAG that are approximately 0.5 mm apart: (i) rostral, (ii) medial and (iii) caudal. AP = anterior-posterior coordinates from bregma. Data are mean ± S.E.M. Using Kruskal-Wallis with Dunn's multiple comparisons tests, * indicates $P<0.05$ comparing sham with 6-OHDA and # indicates $P<0.05$ for 6-OHDA ipsi- vs. contra-latera sides of the brain. (**b**) Quantification of data and representative images of sham and 6-OHDA-PAG lesioned rats measuring mean TH^+ cell numbers in the (i-iii) VTA and (iv-vi) DRN, and TPH^+ cell numbers in the (vii-ix) PAG and (x-xii) DRN. Data are mean ± S.E.M and were analysed using an unpaired t test.

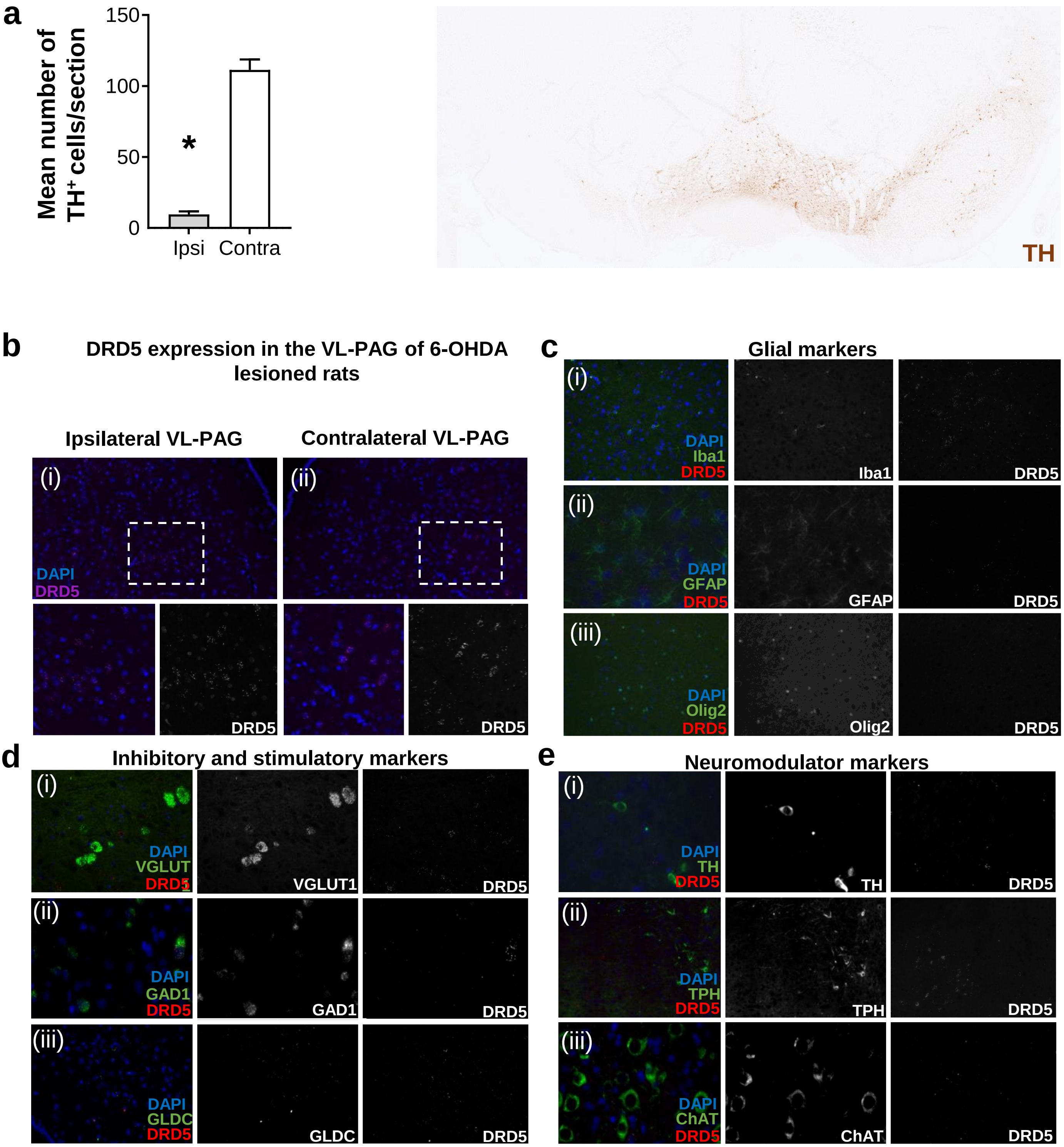

Supplementary Figure 4: Confirmation of TH$^+$ cell loss in the SNc and colocalization of neural cell types with DRD5 in the VL-PAG. (**a**) Comparison of TH$^+$ cell numbers in the SNc of 6-OHDA lesioned rats in the ipsi- and contra-lateral hemispheres with a representative image of ipsilateral (left) TH$^+$ loss in 6-OHDA lesioned rats compared to contralateral (right) side. (**b-e**) Representative images showing PAG-localised DRD5 expression; (**b**) in the VL-PAG of 6-OHDA MFB lesioned rats; (**c**) with glial markers; Iba1 (i), GFAP (ii), and Olig2 (iii), (**d**) inhibitory and stimulatory neuronal markers; VGLUT1 (i), GAD1 (ii), GLDC (iii), and (**e**) neuromodulator markers; TH (i), TPH (ii), and ChAT (iii). Greyscale images are also shown of each individual marker and DRD5 expression within the same area. Data are mean ± S.E.M. * indicates $P<0.05$ using an unpaired t test.

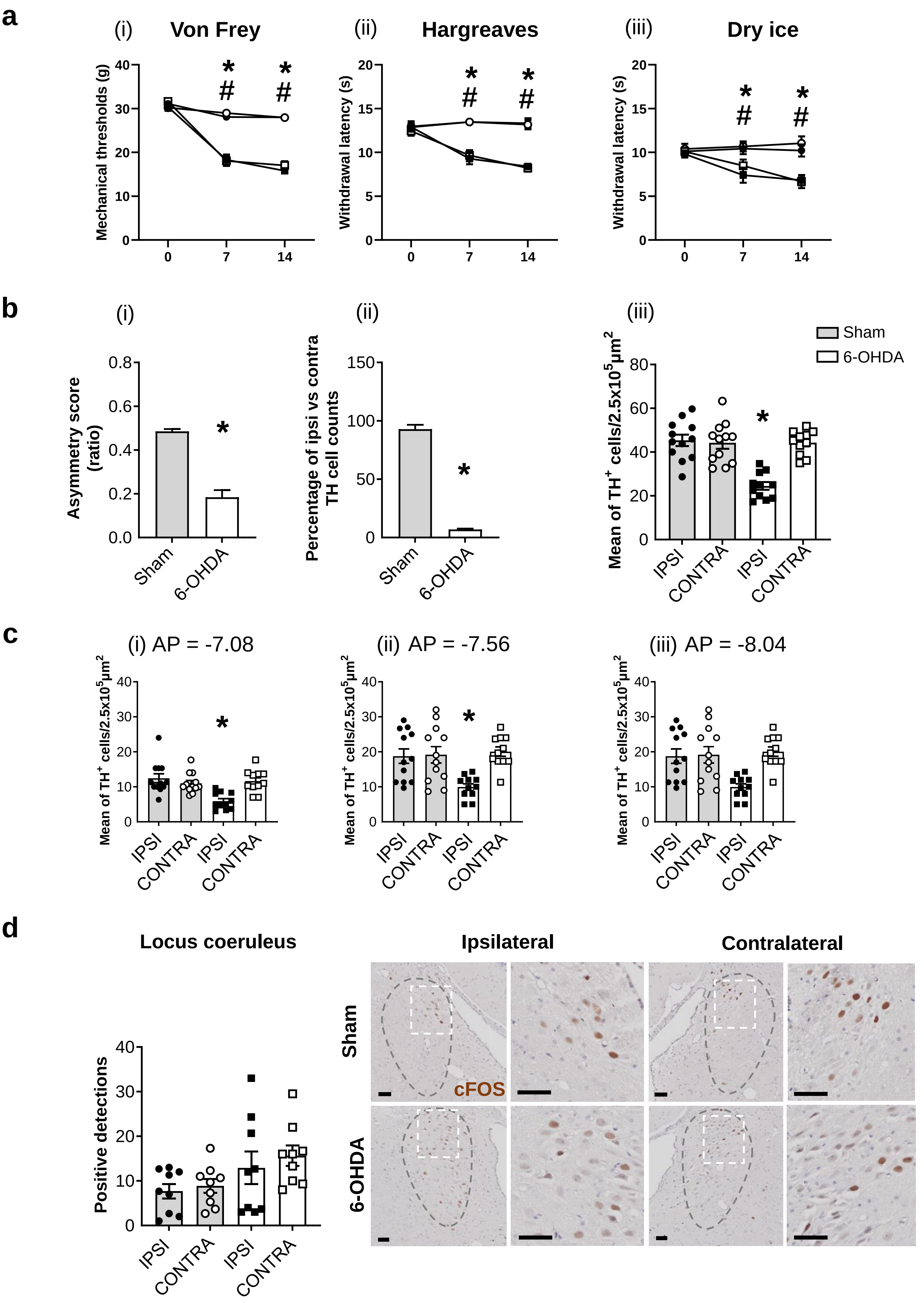
a
(i) Von Frey
Mechanical thresholds (g)
(ii) Hargreaves
Withdrawal latency (s)
(iii) Dry ice
Withdrawal latency (s)
b
(i)
Asymmetry score (ratio)
Sham
6-OHDA
(ii)
Percentage of ipsi vs contra TH cell counts
(iii)
Mean of TH+ cells/2.5x10^5 µm^2
IPSI
CONTRA
c
(i) AP = -7.08
(ii) AP = -7.56
(iii) AP = -8.04
d
Locus coeruleus
Positive detections
Ipsilateral
Contralateral
cFOS

Supplementary Figure 5: Pronociception, akinesia, and PAG TH^+ loss in hemiparkinsonian rats, with LC c-FOS *post* intra-plantar capsaicin. (**a**) Mechanical and thermal thresholds of sham and 6-OHDA PAG lesioned rats at baseline, day 7, and day 14 post-lesion. (i) Mechanical thresholds (ipsi, $F = 81.28$, $P < 0.0001$; contra, $F = 86.89$, $P < 0.0001$), (ii) heat thresholds (ipsi, $F = 69.94$, $P < 0.0001$; contra, $F = 65.37$, $P < 0.0001$), and (iii) cold thresholds (ipsi, $F = 17.94$, $P = 0.0003$; contra, $F = 14.59$, $P = 0.0009$)were measured using the von Frey, Hargreaves, and dry ice tests, respectively. (**b**) Asymmetry score (i), percentage of ipsi- vs. contra-lateral TH^+ cells in the SNc (ii) and mean number of TH^+ cells detected in the VL-PAG across all 3 regions (iii). (**c**) Mean number of TH^+ cells in each of the 3 different areas of the VL-PAG that are approximately 0.5 mm apart (i) rostral, (ii) medial and (iii) caudal. AP = anterior-posterior coordinates from bregma. (**d**) Number of $cFOS^+$ cell detections in the LC. Representative images showing $cFOS^+$ detections in the LC of sham and 6-OHDA lesioned rats with higher magnification inserts on the right side of each panel. Data are mean ± S.E.M. For nociceptive tests, using two-way repeated measures ANOVA with a Tukey's multiple comparisons test, * indicates $P<0.05$ for contralateral paw responses and # indicates $P<0.05$ for ipsilateral paw responses between sham and 6-OHDA lesion rats. For asymmetry score and percentage of ipsi- vs. contra-lateral TH+ cells in the SNc * indicates $P<0.05$ between sham and 6-OHDA lesioned rats using an unpaired t-test. For histological analyses in the PAG and LC, using Kruskal-Wallis with Dunn's multiple comparisons tests, * indicates $P<0.05$ for sham vs. 6-OHDA ipsilateral measurements, and # indicates $P<0.05$ for 6-OHDA ipsi- vs. contra-lateral sides of the brain. All scalebars = 50 µm.

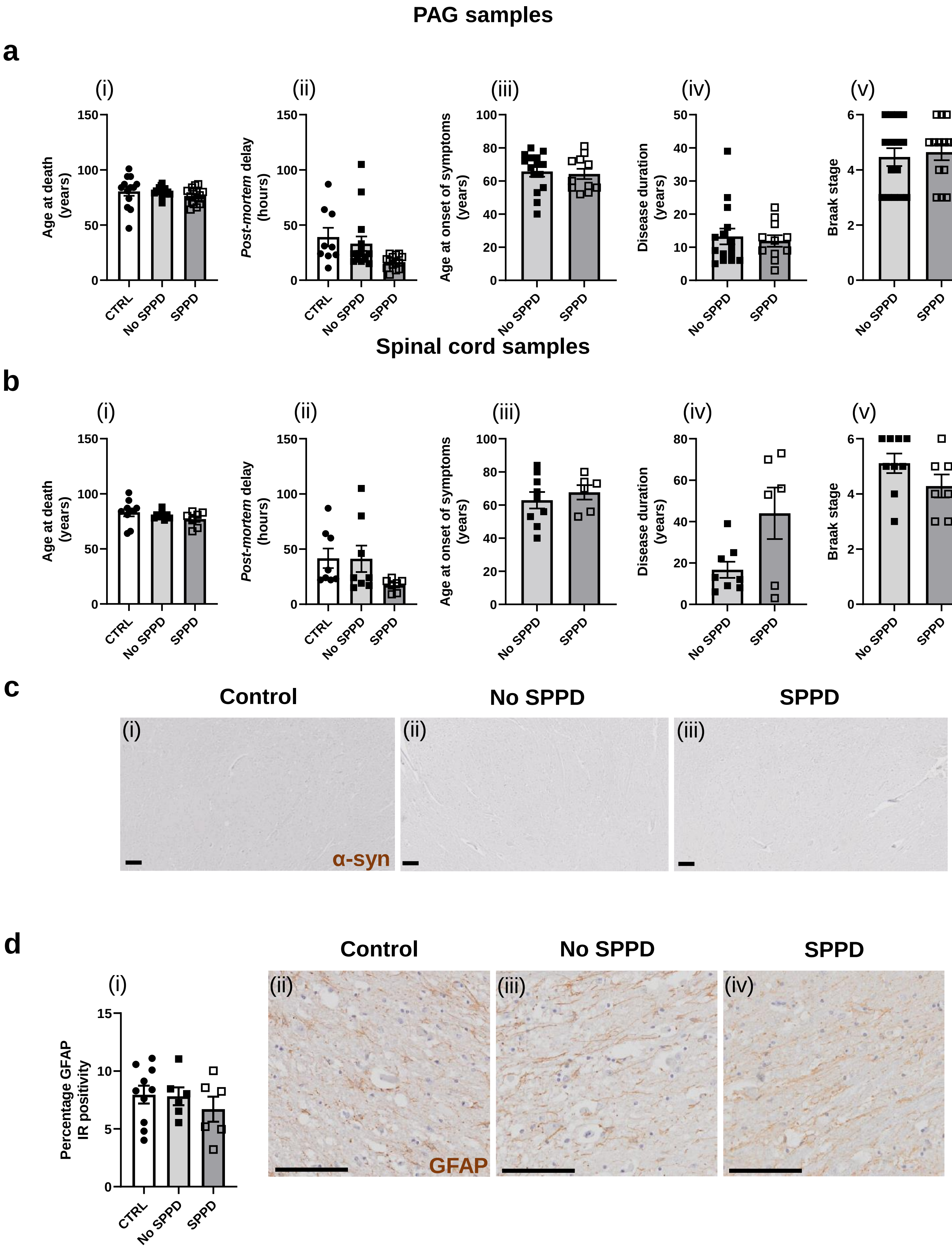
PAG samples
a
(i)
Age at death (years)
150
100
50
0
CTRL
No SPPD
SPPD
(ii)
Post-mortem delay (hours)
(iii)
Age at onset of symptoms (years)
100
80
60
40
20
(iv)
Disease duration (years)
(v)
Braak stage
6
4
2
Spinal cord samples
b
(i)
(ii)
(iii)
(iv)
(v)
c
Control
No SPPD
SPPD
(i)
(ii)
(iii)
α-syn
d
(i)
Percentage GFAP IR positivity
15
10
5
0
(ii)
(iii)
(iv)
GFAP

Supplementary Figure 6: Demographic comparison of *post-mortem* human samples with no Lewy pathology or astrogliosis in the spinal cord. Comparison of (i) age at death, (ii) post-mortem delay, (iii) age at onset of symptoms, (iv) disease duration, and (v) Braak stages for control (CTRL) and Parkinson's disease samples exhibiting spontaneous pain (SPPD) or not (No SPPD) for (**a**) PAG samples (CTRL, n = 12; No SPPD, n = 15; SPPD, n = 11) and (**b**) spinal cord samples (CTRL, n = 10; No SPPD, n = 9; SPPD, n = 7). (**c**) Representative image of an immunostain for α-synuclein Lewy bodies in the dorsal horn of (i) control (ii) No SPPD and (iii) SPPD samples. (**d**) Comparison of GFAP immunoreactivity in the dorsal horn of (i) control (ii) No SPPD and (iii) SPPD. All scale bars = 100μm and data are expressed as mean ± SEM. Analysis of age at death, post-mortem delay and GFAP intensity were performed using Kruskal-Wallis with Dunn's multiple tests. All other analyses were performed using the Mann-Whitney test.

Supplementary Table 1: Co-expression of DRD5 with neural cell types in the VL-PAG. The mean numbers of each cell group detected in the VL-PAG of male Wistar rats (*n* = 3) in a 500μm^2 area. The table also shows number of DRD5$^+$ detections made and whether they are co-expressed with the cellular markers. The markers listed are for neurons (HuC/D), astrocytes (GFAP), microglia (Iba1), and oligodendrocytes (Olig2). Further neuronal subtypes are identified by specific markers: glutamatergic (VGLUT1 and 2); GABA-ergic (GAD1); glycinergic (GLDC); dopaminergic (TH); serotonergic (TPH); and cholinergic (ChAT). N/D = not detected.

Cell marker	Mean number of cells counted ($x \pm$ SEM)	Mean number of DRD5$^+$ cells ($x \pm$ SEM)	Colocalised cells ($x \pm$ SEM)
**HuC/D**	111.8 ± 15.4	41.6 ± 7.1	37.3 ± 6.7
**GFAP**	25.6 ± 2.3	13.3 ± 1.2	N/D
**Iba1**	16.7 ± 1.2	33.3 ± 5.8	N/D
**Olig2**	32.0 ± 2.0	27.7 ± 4.2	N/D
**VGLUT2**	50.3 ± 2.0	23.4 ± 2.2	18.6 ± 1.7
**VGLUT1**	9.0 ± 0.6	10.2 ± 0.6	N/D
**GAD1**	21.9 ± 3.9	10.6 ± 2.9	1.9 ± 1.1
**GLDC**	3 ± 1.0	18.8 ± 4.9	N/D
**TH**	7.2 ± 0.2	5.9 ± 0.8	0.3 ± 0.1
**TPH**	5.9 ± 0.9	4.5 ± 0.5	0.1 ± 0.1
**ChAT**	7.9 ± 2.3	2.6 ± 0.7	N/D

Supplementary Table 2: Cohort and clinical demographics. A list of the *post-mortem* samples Parkinson's disease (PD) or control studied detailing their clinical diagnosis, Braak stage, the age at disease onset, age at death, disease duration, sex, and *post-mortem* delay. Additionally listed is whether periaqueductal grey (PAG), spinal cord (SC) or both tissues were used from each patient and whether the patient was reported as exhibiting spontaneous pain or not in clinical notes.

Case number	Clinical diagnosis	Braak stage (1-6)	Age at disease onset	Age at death	Disease duration	Sex	Post mortem delay (h)	Tissue sample	Clinical report of pain (Burning, tingling, general pain)
1	Control	-	-	84	-	F	22	BOTH	-
2	Control	-	-	87	-	M	31	BOTH	-
3	Control	-	-	84	-	F	23	BOTH	-
4	Control	-	-	81	-	F	22	SC	-
5	Control	-	-	94	-	F	24	BOTH	-
6	Control	-	-	94	-	M	11	PAG	-
7	Control	-	-	101	-	M	60	BOTH	-
8	Control	-	-	64	-	M	80	BOTH	-
9	Control	-	-	87	-	M	57	BOTH	-
10	Control	-	-	47	-	F	30	PAG	-
11	Control	-	-	84	-	F	-	BOTH	-
12	Control	-	-	74	-	F	-	PAG	-
13	Control	-	-	66	-	F	-	BOTH	-
14	PD	4	74	83	9	M	24	BOTH	-
15	PD	5	84	-	-	F	-	SC	-
16	PD	-	76	5	81	M	29	PAG	-
17	PD	5	80	88	8	M	15	BOTH	-
18	PD	4	72	82	10	F	19	PAG	-
19	PD	5	53	78	22	M	17	BOTH	-
20	PD	5	68	81	13	M	24	BOTH	-
21	PD	6	70	84	14	F	21	PAG	-
22	PD	6	47	78	31	F	46	BOTH	-
23	PD	6	56	81	25	F	80	BOTH	-
24	PD	6	40	79	29	F	105	BOTH	-
25	PD	3	74	80	6	M	24	PAG	-
26	PD	3	64	76	12	F	19	BOTH	-
27	PD	3	64	70	6	F	17	PAG	-
28	PD	3	78	86	8	M	24	PAG	-
29	PD	3	70	86	16	F	33	PAG	-
30	PD	6	60	69	9	M	9	PAG	Back pain before tremor
31	PD	6	57	74	17	M	14	PAG	Neuropathic pain
32	PD	5	52	64	12	M	5	PAG	Burning feet at night
33	PD	5	56	75	19	M	11	PAG	Pain in thighs and lower back
34	PD	4	72	81	3	F	24	PAG	Neck & Shoulder pain
35	PD	3	74	83	9	F	9	SC	Chest pain
36	PD	4	77	80	3	F	21	BOTH	Lower back & abdominal pain
37	PD	5	81	87	6	M	16	PAG	Intermittent pain in left hip
38	PD	4	62	66	4	M	17	SC	Pain in right shoulder
39	PD	5	73	81	8	F	21	BOTH	Worsening lower back pain
40	PD	5	53	76	22	F	10	BOTH	Painful off periods
42	PD	5	71	84	13	M	19	BOTH	Pain in lower back
43	PD	3	56	69	13	M	24	BOTH	Pain in left leg

Supplementary Table 3: Antibodies. List of primary and secondary antibodies used, including supplier, catalogue number, host species for the antibody, and dilution factor. Additional notes include variations in methodology for successful immunostaining from the standard citrate buffer (pH 6.4) heat-induced epitope retrieval (HIER) method. HCOOH = formic acid.

	Antibodies	Company (Catalogue number)	Host	Dilution	Additional notes
Primary antibodies	**c-FOS**	Abcam (ab214672)	Rb	1:4000	
	**CGRP**	Abcam (ab22560)	Sh	1:2000	
	**ChAT**	Milipore (ab144P)	G	1:200	
	**DRD1**	Abcam (ab78021)	Ms	1:250	
	**GFAP**	DAKO (Z0334)	Rb	1:2000	
	**HuC/D**	ThermoFisher (A-21272)	Ms	1:500	
	**Iba1**	Abcam (ab5076)	G	1:1500	
	**Met-ENK**	Abcam (Ab22620)	Rb	1:4000	HIER: TRIS with EDTA (pH 9.0)
	**NeuN**	Milipore (MAB337)	Ms	1:500	
	**NF200**	Abcam (ab82259)	Ms	1:100	
	**TH**	Milipore (ab152)	Rb	1:750	
	**TPH2**	Santa Cruz (sc-48955)	Sh	1:1000	Enhancement with imidazoline (1 mM)
	**α-syn**	Abcam (ab1903)	Ms	1:10000	HCOOH step post-HIER for aggregates
	**β-DH**	Abcam (ab209487)	Rb	1:1000	
	**Olig2**	Sigma-Aldrich (MABN50)	Ms	1:1000	
	**DYN**	Abcam (ab82509)	Rb	1:5000	
	**GAD65/67**	Abcam (ab183999)	Rb	1:8000	
	**GLDC**	Sigma-Aldrich (HPA002318)	Rb	1:1000	
Secondary antibodies	**Anti-Rb (HRP)**	Invitrogen (#31460)		1:200	
	**Anti-Ms (HRP)**	Invitrogen (#31430)		1:200	
	**Anti-G (HRP)**	Invitrogen (#31402)		1:200	
	**Anti-Sh (HRP)**	Invitrogen (#31480)		1:200	
	**Anti-Ms Alexa 488**	Invitrogen (A-11001)		1:1000	
	**Anti-Sh Alexa 488**	Invitrogen (A11015)		1:1000	
	**Anti-G Alexa 488**	Invitrogen (A-11055)		1:1000	
	**Anti-Rb Alexa 488**	Invitrogen (A-11008)		1:1000	
	**Anti-Rb Alexa 594**	Invitrogen (A-11037)		1:1000	

Supplementary antibody data. All antibodies were internally orthogonally validated via protein expression using IHC with comparison to publicly available RNA-seq data of corresponding target in high and low expression tissues. HPA002318 was also orthogonally validated by the manufacturers via protein expression using IHC followed by a comparison to RNA-seq data of corresponding target in high and low expression tissues from the Human Protein Atlas. Previous publications validating the use of the other antibodies are listed below; Ab22560: Rice, F. L., et al. (2019). The evolution and multi-molecular properties of NF1 cutaneous neurofibromas originating from C-fiber sensory endings and terminal Schwann cells at normal sites of sensory terminations in the skin. PloS one, 14(5), e0216527. https://doi.org/10.1371/journal.pone.0216527; Ab78021: Lyu S et al. (2020). BTBD9 and dopaminergic dysfunction in the pathogenesis of restless legs syndrome. Brain Struct Funct 225:1743-1760; Z0334: DeMarino, C. et al. (2023). Detection of SARS-CoV-2 Nucleocapsid and Microvascular Disease in the Brain: A Case Report. Neurology, 100(13), 624–628. https://doi.org/10.1212/WNL.0000000000201682; A-21272: Boyer, L et al. (2005). Myenteric plexus injury and apoptosis in experimental colitis. Autonomic neuroscience : basic & clinical, 117(1), 41–53. https://doi.org/10.1016/j.autneu.2004.10.006; Ab5076: Ma, J. et al. (2021). EphA1 Activation Induces Neuropathological Changes in a Mouse Model of Parkinson's Disease Through the CXCL12/CXCR4 Signaling Pathway. Molecular neurobiology, 58(3), 913–925. https://doi.org/10.1007/s12035-020-02122-x; Sc-48955: Omenetti, A., et al. 2010. Paracrine modulation of cholangiocyte serotonin sythesis orchestrates biliary remodeling in adults. Am. J. Physiol. Gastrointest. Liver Physiol. 300: G303-G315; Ab1903: Recombinant full length protein corresponding to Human Alpha-synuclein. Bengoa-Vergniory, N. et al. (2020). CLR01 protects dopaminergic neurons in vitro and in mouse models of Parkinson's disease. Nat. Comms, 11(1), 4885. https://doi.org/10.1038/s41467-020-18689-x; Ab144P: Jeong, J. H et al. (2015). Cholinergic neurons in the dorsomedial hypothalamus regulate mouse brown adipose tissue metabolism. Molecular metabolism, 4(6), 483–492. https://doi.org/10.1016/j.molmet.2015.03.006; Ab22620: Lau, B. K., Ambrose, B. P., Thomas, C. S., Qiao, M., & Borgland, S. L. (2020). Mu-Opioids Suppress GABAergic Synaptic Transmission onto Orbitofrontal Cortex Pyramidal Neurons with Subregional Selectivity. The Journal of neuroscience : the official journal of the Society for Neuroscience, 40(31), 5894–5907. https://doi.org/10.1523/JNEUROSCI.2049-19.2020; MAB337: Cyr, M. et al. (2006). Dopamine enhances motor and neuropathological consequences of polyglutamine expanded huntingtin. FASEB journal : official publication of the Federation of American Societies for Experimental Biology, 20(14), 2541–2543. https://doi.org/10.1096/fj.06-6533fje; Ab82259: Awad, H. et al. (2021). Endovascular repair and open repair surgery of thoraco-abdominal aortic aneurysms cause drastically different types of spinal cord injury. Scientific reports, 11(1), 7834. https://doi.org/10.1038/s41598-021-87324-6; Ab152: Goff, L. A. et al. (2015). Spatiotemporal expression and transcriptional perturbations by long noncoding RNAs in the mouse brain. Proceedings of the National Academy of Sciences of the United States of America, 112(22), 6855–6862. https://doi.org/10.1073/pnas.1411263112; Ab209487: Kimura N. (2021). Dopamine β-hydroxylase: An Essential and Optimal Immunohistochemical Marker for Pheochromocytoma and Sympathetic Paraganglioma. Endocrine pathology, 32(2), 258–261. https://doi.org/10.1007/s12022-020-09655-w; MABN50: McQueen, J. et al. (2014). Restoration of oligodendrocyte pools in a mouse model of chronic cerebral hypoperfusion. PloS one, 9(2), e87227. https://doi.org/10.1371/journal.pone.0087227; Ab82509: McAlpine, C. S. et al. (2019). Sleep modulates haematopoiesis and protects against atherosclerosis. Nature, 566(7744), 383–387. https://doi.org/10.1038/s41586-019-0948-2; Ab183999: Augustine, V. et al. (2019). Temporally and Spatially Distinct Thirst Satiation Signals. Neuron, 103(2), 242–249.e4. https://doi.org/10.1016/j.neuron.2019.04.039.
